# Phylogeography and ecological niche modeling unravel the evolutionary history of the Yarkand hare, *Lepus yarkandensis* (Mammalia: Leporidae), through the Quaternary

**DOI:** 10.1186/s12862-019-1426-z

**Published:** 2019-06-01

**Authors:** Brawin Kumar, Jilong Cheng, Deyan Ge, Lin Xia, Qisen Yang

**Affiliations:** 1 Key Laboratory of Zoological Systematics and Evolution, Institute of Zoology, Chinese Academy of Sciences, 1 Beichen West Road, Chaoyang District, Beijing, 100101 People’s Republic of China; 2 International College, University of Chinese Academy of Sciences, Beijing, People’s Republic of China

**Keywords:** Arid Northwest China, Climate oscillations, Historical demography, Oasis change, Range shift, Taklimakan Desert, Yarkand hare

## Abstract

**Background:**

The Taklimakan Desert in China is characterized by unique geological and historical dynamics and endemic flora and fauna, but the influence of historical climate oscillations on the evolutionary history of endemic animals is poorly understood. *Lepus yarkandensis* is an oases-dependent Near Threatened species that lives in fragmented oasis habitats in the Taklimakan Desert, China. We investigated the geological and climatic impacts on its geographical differentiation, demographic history and influence of Pleistocene glacial-interglacial cycles on the evolutionary history of *L. yarkandensis*. Further, studied the impact of climatic oscillation based modification on phylogeography, distribution and diversification pattern of Yarkand hare by using Cytb (1140 bp), MGF (592 bp) and SPTBN1 (619 bp) markers. Ecological niche modeling (ENM) revealed the evolutionary history of this species in response to climate change during the Quaternary. Paleodistribution modeling was used to identify putative refugia and estimate their historical distributions.

**Results:**

Both historical demographic analyses and climatic niche modeling revealed strong effects of glacial climate changes, suggesting recurrent range contractions and expansions. The EBSP results indicated clear population expansion of *L. yarkandensis* since the Pleistocene. In the “early Pleistocene”, the demographic expansion continued from 0.83 MYA to the last glacial period. The ENM analysis supported a wide distribution of *Lepus yarkandensis* at high altitudes during the last interglacial (LIG) period. During the last glacial maximum (LGM), the suitable climate was reduced and restricted to the western part of the Taklimakan Desert.

**Conclusions:**

Inland aridification, oasis evolution and river flow played major roles in the population differentiation and demographic history of Yarkand hares. Historically, the large, continuous oases in the Taklimakan Desert contained a viable and unique population of *L. yarkandensis.* The fragmented desert environment might have caused low gene flow between individuals or groups, thus leading to predominant genetic differentiation. The Pleistocene climatic cycles triggered the diversification and expansion of this species during cold and warm periods, respectively, leading to multiple colonization events within the Taklimakan Desert. These events might be due to the expansion of the Taklimakan Desert during the Middle Pleistocene. Yarkand hare previously occupied vast areas at low and intermediate altitudes in Xinjiang, Gansu, Shanxi, Henan and Shaanxi Provinces in China. The past aridification, climate change-induced oasis modifications, changes in river volumes and flow directions, and human activities all affected the population demography and phylogeography of the Yarkand hare.

**Electronic supplementary material:**

The online version of this article (10.1186/s12862-019-1426-z) contains supplementary material, which is available to authorized users.

## Background

The climate at least partially controls the structure and function of essential terrestrial ecosystems, particularly in dry environments [1–3]. Knowledge of vegetation dynamics and water cycles is crucial for predicting the variability of climate and water resources in water-limited landscapes, such as deserts [4, 5]. Desert regions are extremely sensitive to climate change, which is linked to the spatiotemporal heterogeneity of heat and precipitation that causes environmental changes in deserts and their oases [6]. Oases are medium- or small-sized, arid and semiarid regions supported by natural rivers in deserts with mesophytic or xeromesophytic plants [7]. Therefore, oases range dynamics and range shifts in organisms are directly linked to the presence of water [6], which is sensitive to global climate change [7]. In addition, oases always have a stronger impact than the regional climate on physiological processes of animals and local species richness [8–10]. Recent evidence has showcased the effect of climate change on the range shifts of plant and animal species poleward and to higher elevations based on a field study from lowlands to the upper subalpine vegetation belt (0 to 2600 m above sea level), spatial species datasets and integrated modeling approaches [11–13]. Recent studies suggest that the temperature increased at rates of 0.32–0.36 °C/decade and 0.1–0.3 °C/decade in the arid regions of Northwest China and the Tarim Basin, respectively, over the last 60 years [14]. However, there has been limited research on the relationship between changes in desert oases and the responses of species in terms of distribution and demography under historical and current climate changes.

Geological and historical events associated with Pleistocene climate change, are one of the most important historical process influencing current geographic distribution and genetic variation [15–19]. These remarkable climatic fluctuations during the Quaternary had a strong impact on the distribution, range sizes and genetic diversity of species [20, 21]. The velocity of this late Quaternary glacial-interglacial climate change increased the extinction risks of species [22]. A large number of leporids became extinct after the Pleistocene because of extreme climatic conditions during these Quaternary glacial periods in America (*Aluralagus*, *Lepoides*, *Paranotolagus*, *Pewelagus*, *Notolagus*, *Pratilepus*, *Alilepus*, *Nekrolagus*, *Aztanolagus*, and *Pewelagu*), Eurasia (*Serengetilagus*, *Pilosiwalagus*, *Veterilepus*, *Nuralagus*, *Pliopentalagus*, *Alilepus*, *Tsaganolagus* and *Sericolagus*), Africa (*Alilepus* and *Serengetilagus*) and Asia [23]. During the Pleistocene glaciations, the spread of ice sheets increased the aridity, and the reduced temperature promoted the conditions for allopatric divergence among isolated populations, which led to speciation [20, 24, 25]. The evolutionary processes of species are closely connected to climatic oscillation, aridification, and desert expansion patterns (particularly range contraction and expansion) in arid areas [26]. Knowledge of the influence of past climate changes on organisms is critical when modeling their current and future evolutionary trends [27]. Research on the effects of climatic oscillations on animal population divergence in the arid Taklimakan Desert could improve our understanding of species adaptation and the role of climate change during different periods.

**Fig. 1:**
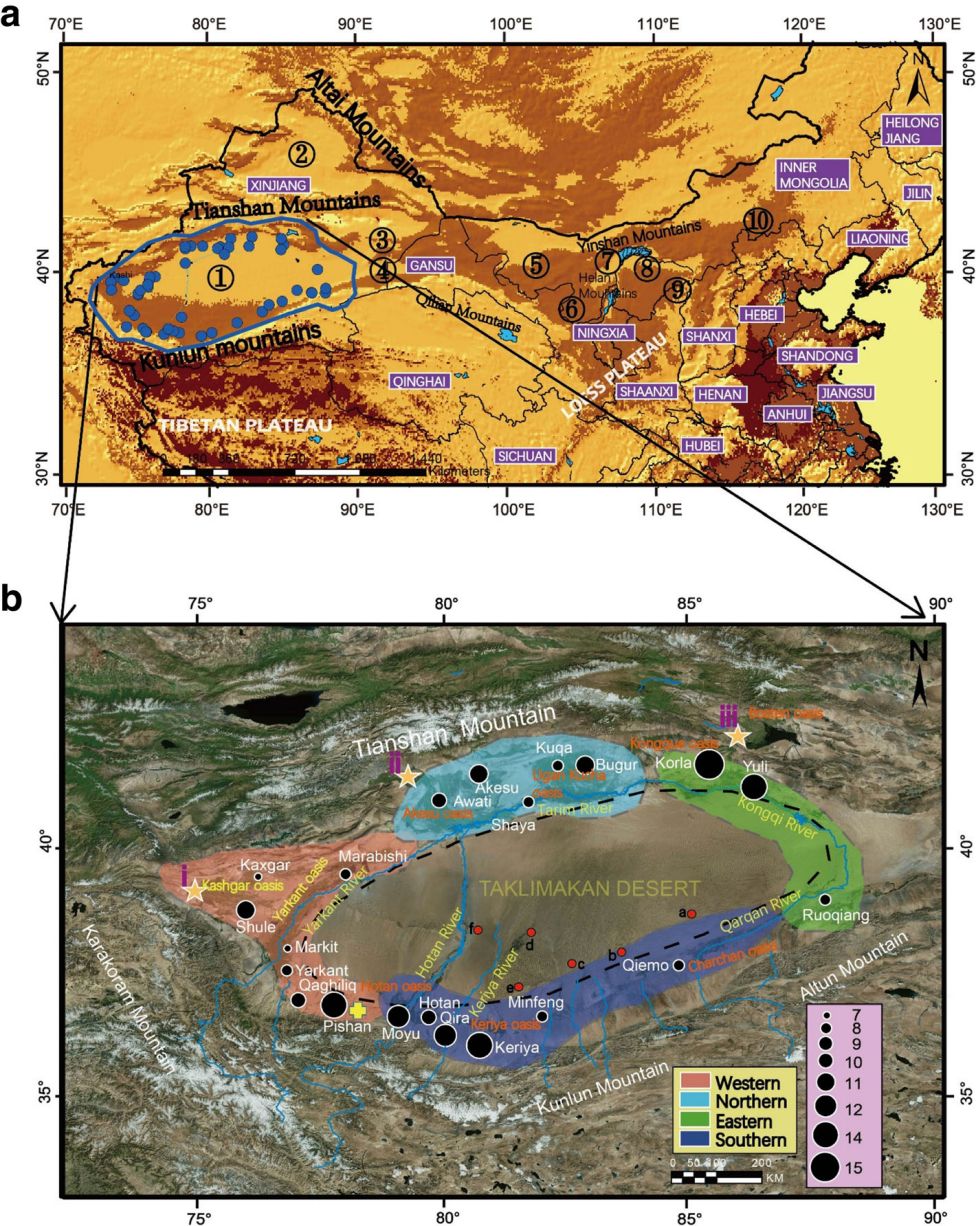
**a** The sandy deserts (active dune fields) and sandy lands (fields of stabilized dunes) in northern China are indicated with numbers.1. Taklimakan 2. Gurbantunggut 3. Kumtag 4. Chaidamu 5. Badain Jaran 6. Tengger 7. Wulanbuhe 8. Kubuqi 9. Maowusu 10. Hunshandake. The blue dots represent the *Lepus yarkandensis* present distribution localities in Taklimakan Desert. The blue line over the desert indicates the geographic range of *L. yarkandensis* based on the IUCN Red List assessment. **b** The distribution of oases and sampling localities of *L. yarkandensis* in Taklimakan desert. The black dots indicating the sampling localities of Yarkand hare. The circle sizes represent the number of samples used in this study. The four sampled populations are coloured as follows: southern = dark blue, eastern = green, northern = ribbon blue and western = orange. The yellow star indicates the locations of *L. yarkandensis* hybridized with the *L. capensis* (i. Shule, ii. Wushi, iii. Yanqi) based on [29]. The ancient (a) Qiemo, (b) Andir, (c) Niya, (d) Karadun, (e) Dandanwulik, (f) Mazatag oasis marked on the map based on [30]. The dashed black lines indicating the oval shaped distribution of Yarkand hares in the desert. The yellow symbol (+) indicates the very old evolutionary history location (Pishan) of *L. yarkandensis* in the Taklimakan desert based on [31]. The map was processed in ArcGIS version 10.2 (ESRI, Redlands, California, USA) (http://www.esri.com/)

The sandy deserts (active dune fields) and sandy lands (fields of stabilized dunes) in northern China are geologically very important landscapes. The Taklimakan Desert is one of the world’s largest and highest deserts and is notable for its large arrays of dune forms, its large bounding alluvial fans, its pluvial lakes, and its ability to produce many dust storms [28]. The Taklimakan Desert is the largest desert in China, with an area of approximately 337,000 km2 and is called the second largest sand sea on Earth. It is located in the center of the Euro-Asian continent and is characterized by a hyper arid, continental climate. Snowfall during winter may affect even the center of the desert. Under present conditions, the desert covers the center areas of the Tarim Basin. The desert is surrounded by Kunlun Shan (Mountains) in the south, the Pamir Plateau in the west, the Tian-Shan (Mountains) in the north and the endorheic lake basin, Lop Nuer, in the east (Fig. 1a). The climate in this area is dry with a mean annual rainfall of less than 200 mm.

Oases are essential components of arid landscapes [32–34], and they are divided into natural oases and artificial oases based on the formation process. (i) Natural oases include desert riparian forests, valley meadows, and shrubs [35, 36]. (ii) Artificial oases develop from natural or desert oases and are influenced by long-term human activities, irrigation, cultivation, artificial landscaping, and habitat degradation [37]. The ancient oases are located not only at the edges of the Taklimakan Desert but also on the riversides and in the center of the Taklimakan Desert such as ancient Qiemo, ancient Andir, Niya, Karadun, Dandanwulik and Mazatag [30] (Fig. 1b). At present, there are seven large and two small oases, i.e., the Bosten (around Bosten Lake), Kongque (along the Kongque River), Ugan-Kucha (along the Ugan-Kucha River), Akesu (along the Akesu River), Kashgar (along the Kashgar River), Yarkant (along the Yarkant River), Hotan (along the Hotan River), Keriya and Charchan oases. The characteristics of the terrain, land cover, climate and hydrology are distinctly different in the mountainous and oasis regions. In the north and west mountainous regions, at altitudes from 2000 to 5400 m, the land cover is mainly wasteland and natural vegetation, while in the alluvial plains, at altitudes from 1000 to 1400 m, irrigated agricultural land is common in many oases. The oasis regions are extremely arid, with annual precipitation less than 50 mm and pan evaporation more than 2100 mm. The river system in the Taklimakan Desert is continuously changing over geological and historical time periods [30, 38]. The Akesu River, which arise from the Tianshan Mountains, the Hotan River come from the Kunlun Mountains and the Yarkant River is bounded by Hotan and Akesu Rivers in the Taklimakan Desert [39]. The Tianshan mountain range is at the north end of the basin, the Kunlun mountain range is to the south, and alluvial plain oasis areas and the Taklimakan Desert are below the mountain range. The western area of the desert consists of fluvial sands and silt [40].

Desertification in this area of the Taklimakan Desert began during the Oligocene-Miocene transition (26.7–22.6 MYA) and has increased since 5.80 MYA, mainly due to the uplift of the Qinghai-Tibet Plateau (QTP) and retreat of the Paratethys [41, 42]. Glaciers developed on peaks of the surrounding mountains [30, 43] and melting snow water flowed along the margin and into the center of the Taklimakan Desert, forming large oases and extensive riparian vegetation belts around the desert [43, 44]. The occurrence of lacustrine deposits in the center of the Taklimakan Desert shows that the area occupied by wetlands was much larger in the past, indicating a more humid environment in the region during the late Quaternary [44]. Earlier studies also suggest that the Taklimakan Desert and the surrounding oases initially formed during the Middle Pleistocene [45]. Glacial-interglacial cycles during the Quaternary caused a series of effects on the basin environments, as the temperatures and water volumes of the rivers increased or decreased due to the fluctuations in the desert and oasis sizes [40, 46]. Variations in the water resources caused by climatic changes were the main factors for the evolution of oases in deserts over geologic time [46]. The sands in the northeast Taklimakan Desert originate from the local Quaternary alluvial plain deposits of the old rivers and lacustrine sediments [47]. Further, the Quaternary environment also produced enormous changes in sand dunes in the northeastern areas [48] and lacustrine deposits from the center and southern margin of the Taklimakan Desert [44]. The Quaternary climatic fluctuations and the changes throughout the landscape could consequently have affected the evolutionary history of the species inhabiting the region.

The Yarkand hare, *Lepus yarkandensis* Günther, 1875, is a drought-tolerant hare endemic to the world’s largest shifting sand desert, the Taklimakan Desert, in Xinjiang, China. They are a small hare with a body weight of 1.4 kg, a body length of 29 to 43 cm, and a tail length of 6 to 11 cm [49]. This species is classified as Near Threatened (NT) on the IUCN Red List of Threatened Species [50]. The Yarkand hare lives in the natural and artificial oasis and edges of the major rivers in the Tarim Basin at altitudes from 900 to 1200 m, further their main distribution is restricted within the riverine patches and scattered oases distributed around the Taklimakan Desert [51, 52] (Fig. 1b). According to a survey conducted by the Xinjiang Forestry Department from 1997 to 2000, the wild Yarkand hare in the Tarim Basin in Xinjiang currently have less than 160,000 individuals. Yarkand hare lives in the poplar forests dominated by *Populus euphratica* (Euphrates poplar) trees and *Salix psammophila* in the desert [49]. Most of the activities of Yarkand hares are associated with the soft sand dunes and willows. Summer is the breeding season for Yarkand hare, and activities of male and female chasing, courtship can last from February to July. The females breed 2 to 3 litters per year [49, 53, 54]. The mating system and social organisation, play significant role on evolution of social behavior in animals [55]. The genus Lepus shows similar social and mating behavior through its different species. In general, most of the species of the genus *Lepus*, have promiscuous mating system [56]. In snow shoe hares (*Lepus americanus*), limited parental care, overlapping home ranges and multiple mating captivity have been noticed by researchers [56, 57]. The mountain hares (*Lepus timidus*) are extremely vagile and commute up to 3 km daily within their home ranges. Interestingly, they do not disperse far in their lifetime [58]. The Tehuantepec jackrabbits (*Lepus flavigularis*) have polygamous mating behavior and non-territorial social organization [59]. Further, home ranges of males are significantly larger than females in breeding season, this clearly indicating a male-biased dispersal in hares [60].

The previous habitats of the Yarkand hare may have been more continuous than their current habitat [61]. The previous research on Yarkand hare revealed that desert features, regional aridification, broad rivers [62, 63], and habitat fragmentation of the desert landscape [61] have all been identified as major factors that influence the genetic structure and act as barriers to the dispersal of the species. There was possibly extensive gene flow among populations during the ancient geological period [61]. Low *SRY* genetic diversity was detected in *L. yarkandensis* [62] and further based on Cyt*b* data: two divergent, geologically different subclades of Yarkand hares have already been found. One subclade was found from the eastern area of the Keriya River, and another was found from the western area of the river [61]. The four populations investigated in this study of the Yarkand hare are accepted according to earlier studies (Fig. 1b; Table 2). The results further revealed high migration rates of *L. yarkandensis* in continuous populations (Northern area –Awati to Akesu) and low migration rates in isolated populations (Western area - Kaxgar to Bugur); No migration was noticed along the Hotan river and old Keriya river [61]. In their study, phylogenetic structures among haplotypes sharing the entire 20 sampling locations, except eastern population (Ruoqiang) in Taklimakan Desert. Further, 24 cranial measurements of Yarkand hares found that, south western population (Pishan) is separated from northern (Korla, Yuli and Shaya) and southern population (Qiemo) [64].

The phylogeny based on *mtDNA* from previous studies does not show any clear phylogeographic structure for Yarkand hares [29, 61]. Based on *mtDNA*, previous researchers used demographical analysis and genetic diversity estimation results to indicate that the western and southern regions might have served as glacial refugia for the Yarkand hare during Quaternary climatic oscillations [31]. Further, 3 postglacial colonization events occurred in the northern and eastern parts of the Taklimakan Desert (0.21, 0.090 and 0.054 MYA), which corresponded to known interglacial periods [31]. These studies also concluded that the western and southern regions of the Taklimakan desert, might contain one or more refugia that were used by the Yarkand hare during times of maximum glaciation. Thus, populations from the southwestern area (Pishan) have a relatively old evolutionary history, and the distribution of the Yarkand hare was restricted to the southwestern parts of the desert during glacial maxima [31].

Here, we combined molecular tools with species distribution modeling to investigate the phylogeographic patterns and evolutionary history of *L. yarkandensis* to unravel the effects of Quaternary climatic oscillations. Specifically, we evaluate (i) whether the low phylogenetic structure in the species occurred because they always had a continuous range, or does it result from postglacial colonizations, which erase any signal of geographic structure? (ii) Did *L. yarkandensis* experience range shifts towards lowland refugia outside the Taklimakan Desert or not? (iii) Does the Taklimakan Desert act as an interglacial refugium and a glacial expansion center for *L. yarkandensis*?

## Results

### Phylogenetic structure and haplotype network

**Fig. 2:**
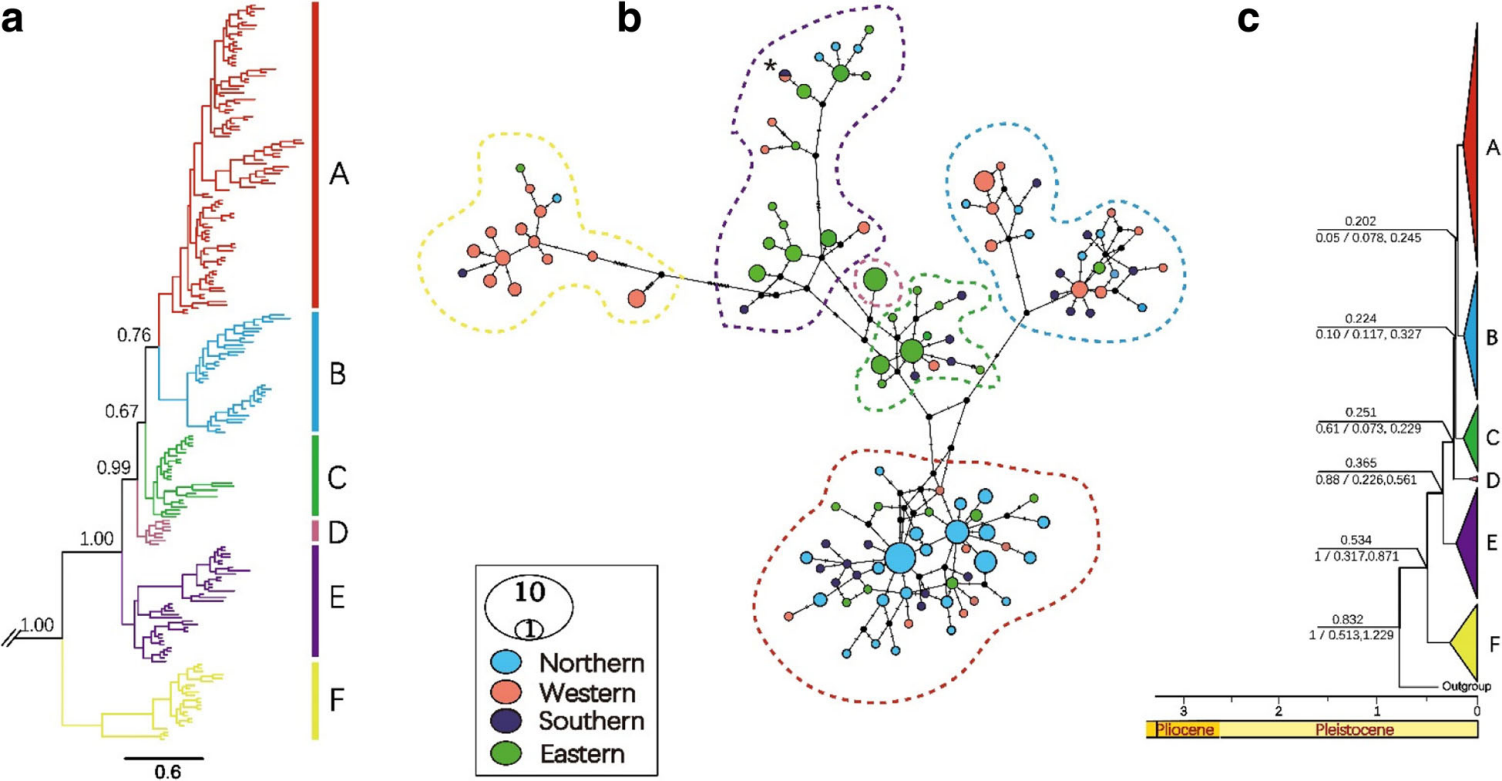
**a** The Phylogenetic tree from the Bayesian inference analysis; the Bayesian posterior probabilities are shown on the branches based on cytochrome *b* (*Cytb*). **b** The Median-joining network of Cytochrome*b* haplotypes for *Lepus yarkandensis*. The circles represent individual haplotypes with size proportional to frequency, branches indicate mutations and black circles indicates hypothetical ancestors. Dots represent undetected haplotypes. The colouring is based on the four populations. The one shared haplotype is indicated with an Asterisk symbol (*). Dashed lines indicate the clades on the haplotype network. **c** Timescale of major divergence among in *Lepus yarkandensis* based on the species tree produced by the *BEAST. The divergence times correspond to the median posterior estimates of their age in MYA. The divergence time estimates and 95% highest posterior density under 0.01 substitutions per million years, and values below branches indicate Bayesian posterior probabilities and HPD. The results are inferred from the *Cytb* data from 219 specimens of *L. yarkandensis*

A total of 219 individuals from 21 localities were sampled from four populations (Southern, Western, Eastern and Northern) in Taklimakan Desert (Fig. 1b. Table 2). The *Cytb* (1140 bp) sequences were successfully sequenced for all the 219 individuals and the *MGF* (592 bp) and *SPTBN*1 (619 bp) genes were sequenced only for 110 of the 219 individuals. The best model according to the BIC was determined to be HKY + I + G for *Cytb*, GTR + G for *MGF* and HKY + G for *SPTBN1*. We found six clades with the BI analyses based on the *Cytb* gene (Fig. 2a). The median-joining networks of *Cytb* showed a poor phylogeographic structure and 118 haplotypes were identified, which did not fully correspond to the sampling populations (Fig. 2b). In addition, shared haplotypes were found between western and southern populations in the desert. The haplogroups corresponding to the sampled populations showed a multiple star-like network together with there are several groups with mutation between them. According to *Cytb* data, he haplotype diversity is very low in southern and eastern population in comparison with northern and western populations. Further the nucleotide diversity is rich in the western population of Yarkand hare (Table 1). A total of twenty haplotypes with low phylogenetic structure were identified from *MGF* and *SPTBN1* markers (Additional file 2: Figure S4 a, b). However, these groups were very similar with the datasets of *Cytb* network results except the number of haplotypes. The network also demonstrated that several haplotypes were very common and shared among many locations. The *mtDNA* indicated that only one haplotype was shared between western and southern populations, but for the *nDNA*, 11 haplotypes from the total of 25 haplotypes were shared among seven locations (Moyu, Akesu, Mingfeng, Marbaishi, Kuqa, Hotan and Korla) (Additional file 2: Figure S4a and b). However, the phylogenetic relationships based on two different nuclear loci were reconstructed with low support due to a slow substitution rate of nuclear genes (Additional file 2: Figure S1 for details). The overall nucleotide diversity was 1.382, with a haplotype diversity of 0.987 based on *Cytb*. The genetic diversity and neutrality test estimates two *nDNA* fragments is given in Table 1. Further, the number of polymorphic (segregating) sites and the raggedness statistic calculated for each gene is mentioned in the Table 1. Further Additional file 1: Table S4 and S5 explaining the genetic variation of Yarkand hares in four population based on *MGF* and *SPTBN1* markers.

**Table 1:** Genetic diversity and neutrality test estimates of *Lepus yarkandensis* based on *Cytb* and *nDNA* for all individuals, each clade and four populations

Loci	All / Clade	n	S	h	Hd	Pi (%)	R Stat	Tajima’s *D*	Fu’s *Fu*
*Cytb*	All / Clade	219	136	118	0.987	1.382	0.002	−0.111	0.304
	A	92	49	46	0.961	0.334	0.027	−0.049	0.048
	B	36	22	24	0.961	0.469	0.026	−0.081	0.219
	C	25	22	14	0.893	0.269	0.035	−0.119	0.338
	D	8	0	1	0	0	0	−0.079	0.239
	E	35	37	21	0.962	0.816	0.018	−0.069	0.23
	F	23	34	15	0.968	0.719	0.032	−0.072	0.367
	Northern population	49	42	34	0.857	0.336	0.037	−0.125	0.223
	Western population	66	47	43	0.943	0.514	0.034	−0.074	0.059
	Southern population	66	54	34	0.521	0.258	0.008	−0.096	0.084
	Eastern population	38	29	21	0.477	0.325	0.017	−0.088	0.192
*MGF*		110	6	13	0.855	0.018	0.055	1.23	−3.425
*SPTBN1*		110	8	7	0.469	0.003	0.15	−1.492	−3.144

The clades, four populations of *Cytb* and two nuclear gene markers summary statistics including *n* number of samples, *S* number of polymorphic sites, *h* number of haplotypes, *Hd* haplotype diversity, *Pi* nucleotide diversity, *R Stat* Raggedness statistic, and the expansion test results for Tajima’s D and Fu’s FS

### Divergence time

A HKY + I + G model of base pair substitution and an uncorrelated relaxed log-normal molecular clock with a prior coalescent tree of constant size were used. *L. yarkandensis* species diversified during the Pleistocene, and the diversification of the species corresponded to the climatic oscillations in this period. The most recent common ancestor of *L. yarkandensis* and the outgroup species (*L. sinensis*, which was the closest species in our tree) occurred approximately 0.832 MYA (95% highest posterior density (HPD): 0.513–1.229) (Fig. 2c). Clade F diverged first from the other clades at approximately 0.534 MYA (95% HPD: 0.317–0.871), placing the ancestry of all living representatives of this species in the Middle Pleistocene. Both the ancestry and the divergence into the different clades took place during the Middle Pleistocene. The E clade appeared approximately 0.365 MYA (95% HPD: 0.226–0.561). Clades C and D also diverged during the Middle Pleistocene, approximately 0.251 MYA (95% HPD: 0.073–0.229) and 0.224 MYA (95% HPD: 0.117–0.327), respectively. Subsequent divergence events occurred starting at the beginning of the same period, with clade B separating approximately 0.202 MYA (95% HPD: 0.078–0.245) (Fig. 2c).

**Fig. 3:**
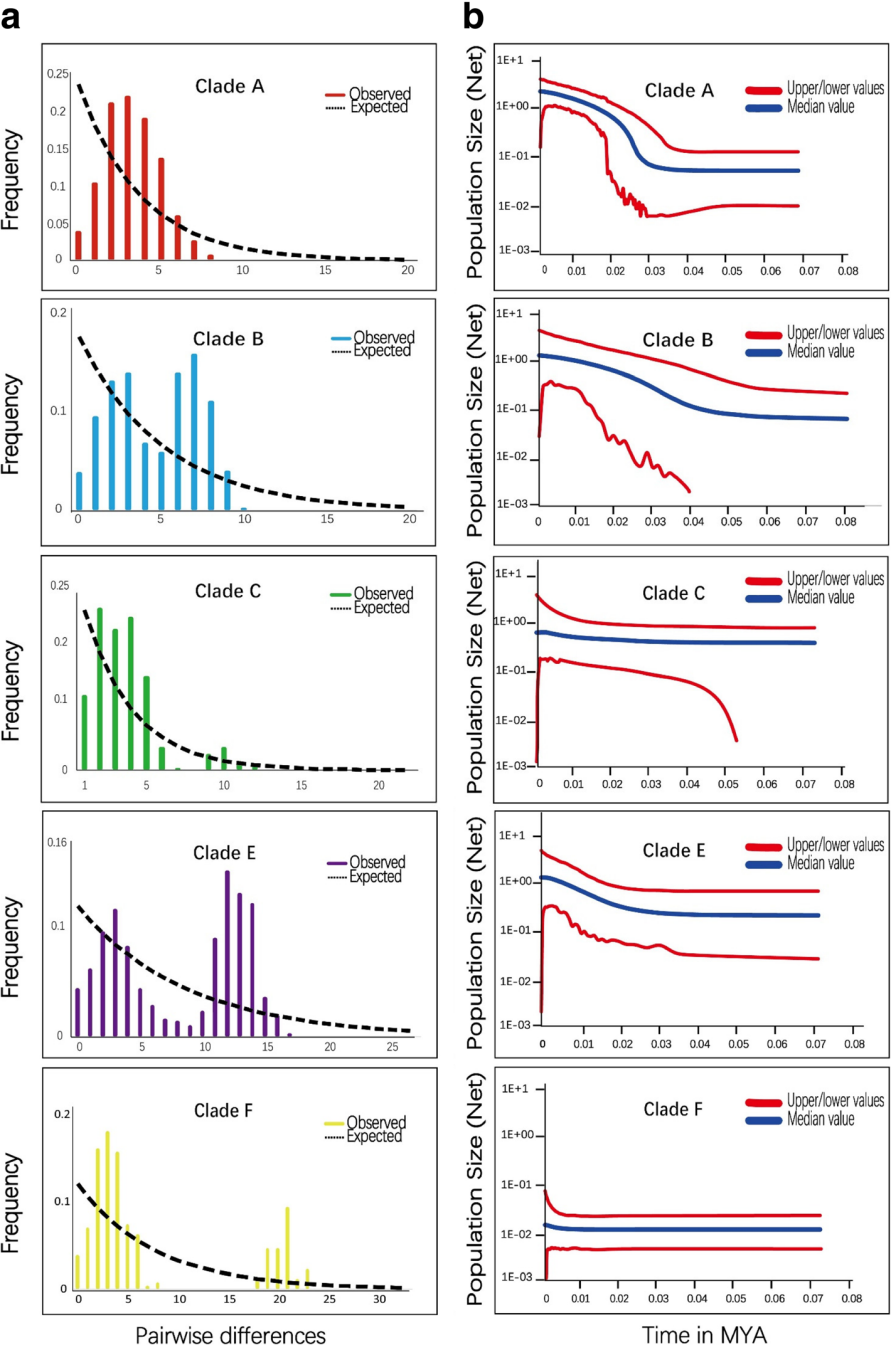
**a** Pairwise mismatch distributions for major clades based on the mtDNA. The coloured bars indicates the observed distribution of pairwise differences, and black dashed lines represent the theoretical expected distribution under a population expansion model. **b** The historical demographic trends represented by Extended Bayesian Skyline Plot (EBSP) based on *Cytb*. The median population size in the blue coloured lines, and the red lines representing the upper and lower 95% confidence intervals. The x-axis of the skyline plots is time in millions of years before the present, and they y–axis is the estimated effective population size (Ne)

### Historical demographic analysis

The analysis was performed for each haplogroup that was defined based on the *Cytb* variation. For the entire data for the six clades showed entirely different results for Tajima’s *D* and Fu’s *Fs* (Table 1). Tajima’s D values are all negative while Fu’s Fs are all positive. The negative values of *D* (*P* ≤ 0.05) indicate that populations are not at equilibrium, possibly due to a recent range expansion or recovery from a population bottleneck [65]. The mismatch distribution analysis revealed that only clade A showed a unimodal curve (Fig. 3a), with the sharp peak indicating a recent demographic expansion of the Yarkand hare. From the 219 individuals, a total of 118 haplotypes based on *mtDNA* including clade A (46), clade B (24), clade C (25), clade D (8), clade E (35) and clade F (23) were retrieved. From the 110 individuals, a total of 20 haplotypes based on nuclear *DNA* were retrieved (Table 1). The pairwise mismatch distributions for total four populations of *Cytb*, nuclear genes *MGF* and *SPTBN1* showed a nonuniform model, indicating a clear expansion (Additional file 2: Figure S3a-c). The demographic scenario for *L. yarkandensis* determined through EBSP analysis suggested different patterns for different clades. Further, the results of the EBSP analysis of the mitochondrial sequences showed profound demographic expansion (Additional file 2: Figure S3d). The two *nDNA* based EBSP analysis described in Additional file 2: Figure S3e and f. The population expansion of clades A, B and E started at the Late Pleistocene (ca. 30–20 thousand years ago) and continued until the present, thus including the Holocene. Clade A showed a sudden population expansion beginning in the upper Pleistocene (Fig. 3b), and clade B showed a gradual expansion during approximately the same period. Clades C and F were stable in terms of the Ne (Fig. 3b). However, the distribution range of clade E expanded during the same period, and those of clades A and B increased considerably. The demographic results based on EBSPs in the present study clearly showed that the expansion of the Yarkand hare population occurred when the climate began to warm (Fig. 3b).

### Species distribution models

**Fig. 4:**
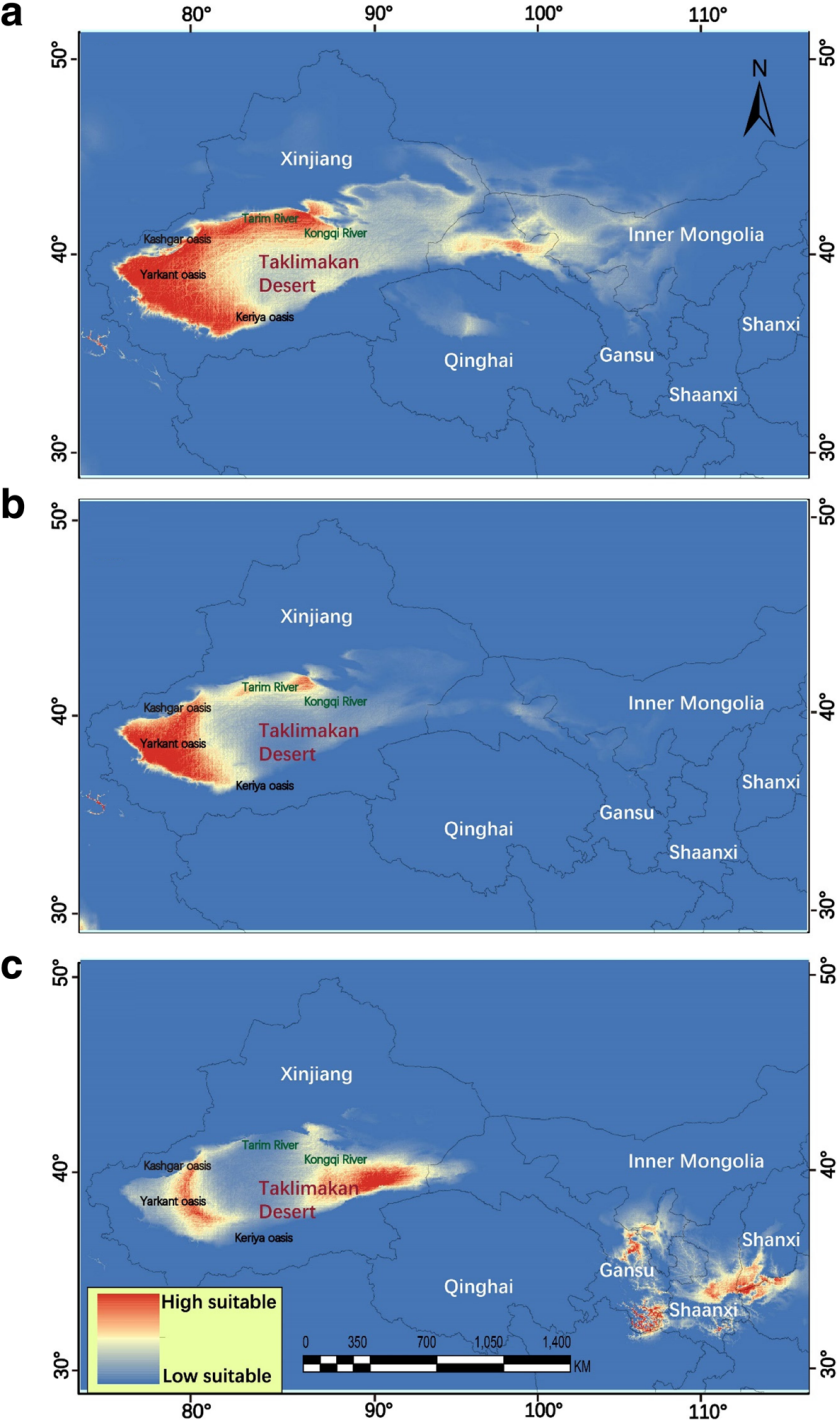
Modelled suitable potential distribution as occurrence probability of *Lepus yarkandensis* in north-western China sandy patches and deserts during different climatic scenarios. The predicted distribution probability was based on the 10% logistic training threshold estimated by Maxent 3.3.3 k. **a** The climatically suitable areas for *L. yarkandensis* during present condition; **b** the last glacial maximum (LGM); **c** the last interglacial (LIG). The high suitable (red) and low suitable (blue) habitats relative to the latter period. All models were developed using the “maximum entropy model” as implemented in the software Maxent 3.3.3 k

The AUC was 0.977 for the model generated for all three periods, which confirmed the excellent predictive power of our models. The binomial probabilities (< 0.001) for ten common thresholds indicated that our predictions were significantly better than those from a random prediction model. The predicted climatically suitable habitat for the Yarkand hare was not completely coincident with the present geographic distribution of the species (Fig. 4a). Taklimakan Desert oases were the major habitats for the present conditions, and a few oases in Gansu Province were also predicted as suitable areas for Yarkand hares (Fig. 4a). The western and northern parts of the Taklimakan Desert have highly suitable habitat based on current environmental conditions. However, a very different pattern was estimated under LGM conditions. The dominant trend of westward contraction into the Taklimakan Desert was predicted (Fig. 4b). The climatically suitable habitat for *L. yarkandensis* consisted of large individual areas distributed in the western parts of the Taklimakan Desert during the LGM. These data clearly showed a potential range shift that impacted the distribution of *L. yarkandensis* between these time periods, as suggested by the differences between the LGM projection and the projections for the other periods (current and LIG periods). Under the modeled climatic conditions of the LIG period, the climatically suitable habitat for *L. yarkandensis* was prominently present in two distinct areas. The first patch that was suitable for Yarkand hares included areas in Shanxi, Shaanxi and Gansu Provinces. The second patch included the eastern edges and western portion of the Taklimakan Desert (Fig. 4c). The ecological niche modeling (ENM) results revealed a historical range shift of the entire suitable habitat from the Loess Plateau to the Tarim Basin during the Middle Pleistocene. Additionally, the analysis of SDMs revealed that presently suitable habitats are larger than those during the LIG and LGM periods.

### Spatial diffusion analysis

**Fig. 5:**
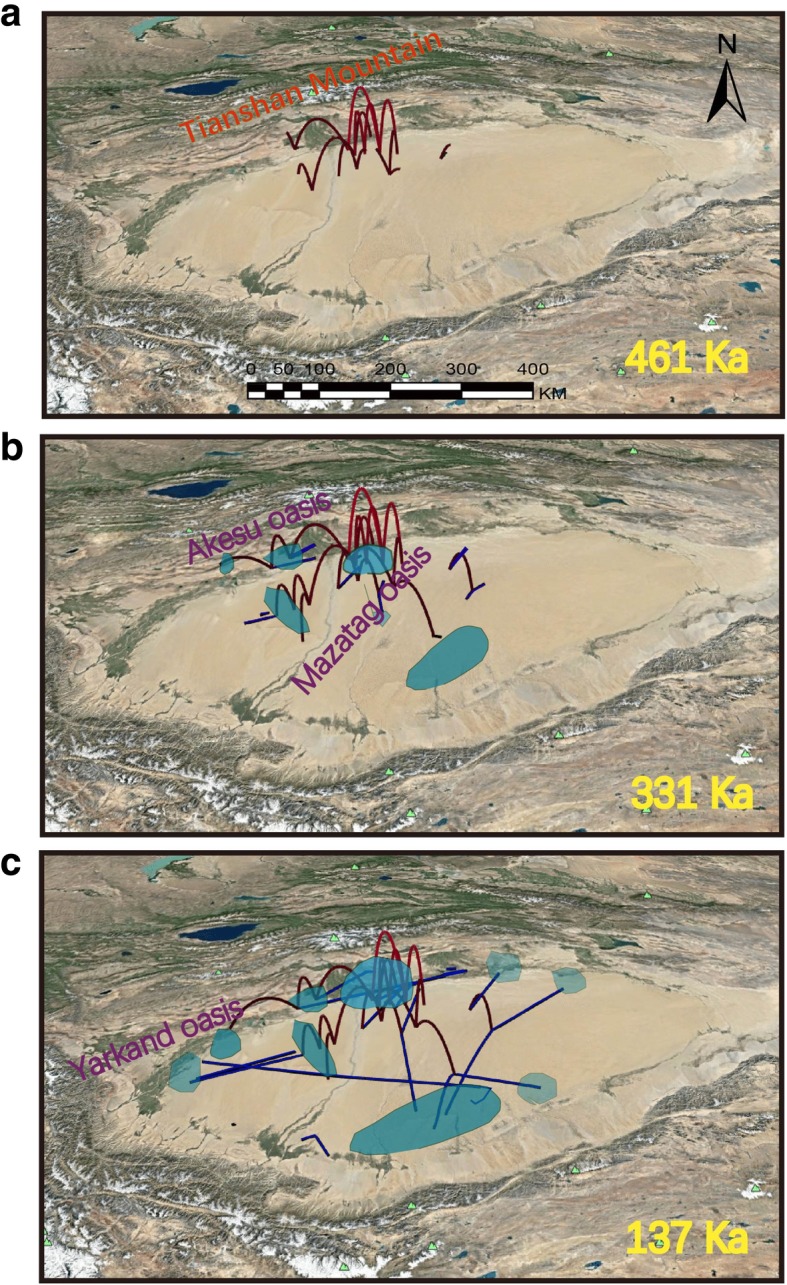
Spatial projection of the diffusion pattern through time, based on the Bayesian phylogeographic analysis in BEAST at three time slices: **a** 461 Ka **b** 331 Ka **c** 137 Ka. The time line was spliced based on the chronology of the Quaternary glaciations in China summarized by [66]. The major rivers indicate in the desert as geological boundaries in population differentiation. The red line indicates the ancient time and the blue line indicates the present time. The brighter polygons represent the early-distributed areas. The map was created by using Google Earth

The phylogeographic diffusion analysis suggested that *L. yarkandensis* originated near the Akesu oasis, which is in the northern part of the Taklimakan Desert. The three clades (central, northwest and southeast) originated from the foothills of the Tianshan Mountains and then likely expanded to the Tarim River oasis, Yarkand River oasis, and Akesu oasis (Fig. 5a). The Akesu oasis area experienced multiple colonization events, and all three clades spread throughout the desert (Fig. 5a). The colonization route appeared inside the Taklimakan Desert 331 Ka (Fig. 5b). *L. yarkandensis* was inferred to have migrated first to the Hotan and Keriya oases in the south of the desert and then to the foothills of the Kunlun Mountains, Kashgar oasis, and Ugan-Kucha oasis during the 137 Ka period, which is the end of the penultimate glaciation period (Fig. 5c). The colonization might have been completed at the end of the last glaciation period.

## Discussion

In this study, we investigated the range-wide phylogeographic structure, diversification and colonization patterns of the oasis-dependent Yarkand hare in the Taklimakan Desert using mitochondrial and nuclear markers. We inferred the demographic history and paleoclimatic conditions of this species to understand its evolutionary history, biogeographic corridors, dispersal routes and diversification. The climatic oscillations had strong relationships with oasis evolution, river flow directions and thus leads to habitat fragmentation. Our results revealed that environmental changes led to the origin of multiple refugia, and the flow direction of the Keriya River later opened pathways for recolonization and led to lineage formation in the southern Taklimakan Desert.

### Origin and diversification of *L. yarkandensis*

In the Late Miocene (12–5.3 MYA), the Tibetan-Himalayan uplift began, which intensely changed the atmospheric circulation in the area and caused widespread aridification across the Tarim Basin [67–69]. These changes triggered the evolution of “increased variability in the East Asia monsoon” [70]. *Lepus* from Eurasia diversified primarily during the Pleistocene (2.5 MYA), and further studies on the general colonization, radiation and historical biogeographical patterns of Lagomorpha suggested that these ancient taxa lived in the forests of Asia [23]. During the Pleistocene, aridification strengthened [71, 72], which may have further promoted the adaptation of *L. yarkandensis* to the desert habitats during this period. Based on the molecular dating in the current study, *L. yarkandensis* originated during the Pleistocene (0.83 MYA) when the deserts in northern China expanded greatly [44]. Pleistocene climate change not only caused range variations and alterations in the Taklimakan Desert but also altered the volume of the rivers [46] and the oasis habitats [73]. The changes in the surrounding mountain environment thus stopped the movement of the species towards mountains and triggered its range shift towards the oasis habitats. Our study is similar with the results of the previous study, that shows the speciation of *L. yarkandensis* was the results of peripheral isolate speciation approximately 0.64 MYA (± 0.26 MYA) [74].

The presence of sandy loess at the southern margin of the Taklimakan Desert indicates that this area was probably not as arid in the past as it is today because the environment associated with sandy loess deposition is generally less arid than the environment that produces sand seas [75]. The Xinjiang loess is mainly distributed on the terraces of rivers, in low uplands, in piedmonts and along the margins of deserts. The thickness of loess varies from tens of meters to hundreds of meters. The Xinjiang loess began developing in the Middle Pleistocene, and the oldest Xinjiang loess was deposited during the early Pliocene [76]. Interestingly, clade F of the Yarkand hare is mainly distributed in the northwestern areas of the Taklimakan Desert. According to the SDM results, the mountains of the Gansu, Shaanxi and Shanxi Provinces could have acted as suitable habitat during the LIG period. The hexi corridor may have served as a migratory corridor for the Yarkand hare during the LIG to LGM period. *P. euphratica* (Euphrates poplar) trees exhibited similar contraction and expansion patterns during the LIG and LGM in China [77]. Further, the phylogeographic diffusion analysis indicated that the ancient clades originated from the foothills of the Tianshan Mountains. These clades followed the expansion routes further west and to the middle of the Taklimakan Desert. Initially, the expansion occurred towards the Tarim River oasis and Yarkand River oasis, and multiple colonization events occurred in the Akesu oasis area (Fig. 5a) during the earlier periods. Later, a recent clade (blue lines) showed a clear expansion towards the Keriya oasis and then the foothills of the Kunlun Mountains, Kashgar oasis, and Ugan-Kucha oasis (Fig. 5c). A previous study on other hares, namely, *Lepus castroviejoi* and *L. corsicanus*, showed that the range of separation was not driven by disruptive adaptation but by fragmentation of favorable habitat [78]. Therefore, niche conservatism, which may have been the reason for the fragmentation, may have driven the initial divergence of Yarkand hare.

### Quaternary climatic oscillations and its influence on Yarkand hares

Glacial history is an important factor that shapes regional diversity patterns [79, 80]. Phylogeographic diffusion models and SDMs have been used to locate glacial refugia and examine the range expansions of animals and plants [81–83]. Previous studies using pollen showed no evidence of glaciation in the desert regions of northwestern China [68, 84, 85]. The Pleistocene climate in the Tianshan Mountains showed a general trend towards enhanced aridity [86]. During the beginning of the Early Pleistocene, the Tianshan Mountains were occupied by a vast sheet of ice, which resulted in the formation of the Tarim River and associated rivers [75, 87, 88]. The present phylogeographic diffusion analysis found a clear pattern in which *L. yarkandensis* dispersal routes were from the foothills of the Tianshan Mountains to the southwestern part of the Taklimakan Desert (Fig. 5c). The populations from lower elevations might have become extinct or subdivided because of fragmentation of suitable environments. The Tarim River oasis and Yarkand River oasis may have been suitable habitats for the Yarkand hare, followed by the Hotan and Keriya oases in the southern part of the desert. Later, the foothills of the Kunlun Mountains, the Kashgar oasis, and the Ugan-Kucha oasis were also used as dispersal routes. The northern low-altitude areas of the Taklimakan Desert were found to be the routes used for multiple colonizations by the Yarkand hare due to the availability of larger oases and sufficient water runoff. These results showed a clear pattern of dispersal from north to southwest in the Taklimakan Desert (Fig. 5c). During later periods, the distribution pattern of *L. yarkandensis* formed in response to a combination of different conditions such as climate and river systems. The Keriya River may act as a barrier between the eastern and western areas of the Tarim Basin for *L. yarkandensis* [61]. The disappearance of glaciers in the Tianshan Mountains at the end of the Pleistocene [89] may have triggered the process of introgression between Yarkand hares and *L. capensis* at high altitudes in the Tianshan Mountains (Additional file 2: Figure S2) [29]. This finding showed that mountain environments are also very suitable for the Yarkand hare, and the process of climate-induced range expansion is ongoing. The Yarkand hare also underwent a severe range contraction, while current anthropogenic impacts such as habitat fragmentation are triggering the isolation of populations and having a substantial influence on the current distribution of the species. Further, during the period of 150Ka, the Taklimakan Desert was arid, and occupied by shifting dunes. At the same time, ice-snow levels increased in mountainous areas, which shows a sudden change in water levels in the Taklimakan Desert [90]. This expansion could have promoted the rapid expansion of Yarkand hares to different oases in the Taklimakan Desert (Fig. 5c).

Presently, the rivers originating from the surrounding mountains, such as the Tarim River and the Kongque River, flow towards the basin (eastwards) under the control of gravity [91]. The sand deposits of the Keriya River (south of the Taklimakan Desert) indicate that the river originated in a glacial environment [73] and acted as a major passage between the northern and southern oases in the Taklimakan Desert [6]. The glaciers reached an altitude of 2500 m in the upper reaches of the Keriya River during the LGM. The extensive distribution of rims created during the last glaciation indicates that there was a considerable amount of water due to deglaciation [73]. During the deglaciation, the rapid melting of glaciers filled the rivers, resulting in ample water in the rivers during the Late Pleistocene, when the climate was more humid than at present [92]. The process described above shaped the flow direction of the Keriya River, and the oasis expansion consequently may have triggered the dispersal of the Yarkand hare towards the southern part of the desert from its northern/western origin. However, during the late period of glaciation, precipitation was higher than it is at present in the southern Taklimakan Desert [75]. Taken together, these patterns indicate that the habitat was very suitable and, thus, rapid colonization occurred within the desert.

At 150 Ka, the vegetation in the oases degraded, and the shifting sand dunes expanded. During these stages, the oases occurred only in the areas where groundwater was relatively plentiful, and the desertification process predominated in the Tarim Basin [92]. This pattern could be a reason for the slow dispersal of the Yarkand hare into only a few southern areas. Since the Late Pleistocene, the oasis evolution in the Tarim Basin has primarily been driven by runoff water flowing into the oases. The variation in runoff and availability of water resources in the oases depended largely on climate change, especially temperature variations. Temperature fluctuations can influence the water resources in oases and cause the oases to evolve. Therefore, climate change was an important factor for oasis evolution in the Tarim Basin throughout geological ages [46].

There was much more precipitation in the Taklimakan Desert approximately 28 Ka than there is now [73]. Due to the extensive snow and glaciation in the Tianshan Mountains, the Yarkand hares could have moved towards the favorable conditions in the desert at low altitudes. At this time, the oasis may have provided very suitable habitat for the Yarkand hares. The oasis expansion could have triggered the movement of Yarkand hares into different parts of the desert. This pattern supports the rapid, aggressive expansion of clade A due to river and oasis modification, which are very favorable for Yarkand hares. The disappearance of vegetation and the formation of aeolian landforms could be the reasons for clade F not reaching the eastern areas of the desert. Further, in the north and west, the large lakes may have prevented the dispersal of Yarkand hares from the northern and southern populations. The past and ongoing climate change triggered modification on the water resources have a major influence on oasis evolution in the Taklimakan Desert [46]. Further, our results also show that the previous distributions of Yarkand hares and their suitable habitat were not only in the Taklimakan Desert but also in nearby sandy lands (Fig. 4c). This all points indicating that, the Yarkand hare distribution is not restricted with in the Taklimakan Desert.

In addition to climate change, human interference is a major problem that influences the distribution of *L. yarkandensis* [61]. The oasis landscape has changed considerably over the last 50 years due to natural flooding and vegetation degradation by human overexploitation [30, 93]. However, due to anthropogenic effects and extensive oasification, the traditional habitats of *L. yarkandensis* no longer host this species, and the phenomenon of habitat fragmentation is irreversible. The shifting sands and the winds already destroyed large areas of the oasis in the southern areas of the desert [30, 94]. The recent studies have noted that the Anthropocene effects have been directly changing the course of the Tarim River [95], the oasis landscape and the vegetation over the last 200 years [30]. The changes in the Kongqi and Tarim Rivers triggered the shrinkage of Loulan oasis (west side of Lop-nur lake), Cele oasis (South) and Yingjisha oases [6]. The rapid habitat degradation has changed the fertile soil into soil with low soil water content, high concentrations of salt and a structureless soil with very low organic matter content in the Yutian-Hotan oases [96]. The smaller Charchan oasis (south) has also undergone severe changes due to the changes in the land-use pattern [97]. The Kashgar oasis receives less rainfall and undergoes extreme temperature fluctuations (50 °C in summer to − 20 °C in winter) [98], which could be unfavorable for the Yarkand hares. The heavy aeolian deposition is known to be one of the most threatening natural hazards to the Qira oases in the Taklimakan Desert [99]. As a transitional belt between the desert and oasis, the ‘ecotone’ plays a major role in the balance of the oasis ecosystem [100]. This role of the ecotone is mainly driven by the amount of its natural vegetation cover. The destruction of vegetation cover due to human activities has been considered the primary cause of environmental degradation in the Tarim Basin [101].

### Demographic expansion under climate change

The *mtDNA* haplotype network is a multiple star-like networks together, mainly consisting of the northern (blue) populations is on one side of the network. Whereas, the *nuDNA* network shows a north-western population have a long evolution history. This indicating a recent population expansion, and these populations might have a strong demographic history. The results also indicated that *L. yarkandensis* in the Taklimakan Desert underwent population growth followed by an apparent demographic expansion during the Pleistocene (Fig. 3b). A previous study found significant signals of potential demographic and range expansions of the Yarkand hare during the Late Pleistocene interglacial period (0.07–0.15 MYA) [61]. The time of demographic expansion of *L. yarkandensis* was estimated to be between 0.175 MYA and 0.171 MYA [29]. Our demographic analysis showed that the demographic expansion started during a glacial period 0.02 MYA. The EBSP analysis found evidence of population expansion started around the end of the last glacial period possibly during the transition to the Holocene (Fig. 3b). Further, extensive gene flow may have occurred among Yarkand hare populations during ancient geological periods [61]. The multiple colonization events in the Akesu oasis and further geographical barriers erased genetic differentiation. The *mtDNA* not always reflect the species’ overall evolutionary history [102]. The main drawbacks are associated with the mtDNA studies are, sex bias and/or introgressive hybridization [103] and genetic drift [104]. The results of *mtDNA* and *nuDNA* markers show different haplotype networks, since the difference in inheritance. The mitochondrial marker results show 6 lineages. These lineages could not be recovered by the nuclear markers, which might be because of reduced signal and incomplete lineage sorting, and/or lack of resolution at these nuclear loci (Additional file 2: Figure S1). The analysis of nuclear data did not produce well resolved with the clades, thus shows hidden gene flow and genetic admixture. The discordance in *mtDNA* and *nuDNA* genes because of recent mitochondrial admixture in Yarkand hares. The maternal linage and the male biased dispersal in Yarkand hares could be also responsible for the genetic differentiation. The genetic variation noticed on all the four populations based on *Cytb* (Table. 1) and other two nuclear markers (Additional file 1: Table S4 and S5) clearly showing the Yarkand hare population differentiation with the desert landscapes are very clear. The long-term changes on population sizes and evidences on stronger population bottlenecks could worsen the situation due to the ongoing hybridization with the *L. capensis* species.

The phylogeographic structure based on *mtDNA* in this study did not show any clear structure as similar with previous studies [29, 61]. Interspecific hybridization could cause the long-term phylogenetic discordance between the nuclear and mitochondrial genes in Yarkand hares. Niche breadth played a role in the life history, demography and diversification of the Yarkand hare population, which were the key ecological determinants in different historical periods. Finally, the Yarkand hares are holding the unique genetically distinct lineage that is older than previously thought. Ongoing impact of current climate change in this desert landscape is likely to be a major threat for Yarkand hares.

## Conclusion

In conclusion, our integrative approach that merged phylogeography and SDMs provided considerable evidence to support the hypothesis that high-altitude organisms were heavily affected by Pleistocene climatic fluctuations. The sandy desert formation process could have played a main role in the development of the oasis and oasis-dependent animals. The ongoing changes to the oasis landscapes and their associated fauna resulting of Pleistocene climatic changes. The geology of the Taklimakan Desert, along with the evolution of the Yarkand hare, was likely shaped by aridification in the past and present. The cyclic increase and decrease in water distribution appear to have allowed for interruptions of gene flow or influenced the evolutionary process. Our results also suggest that Pleistocene and associated evolutionary mechanisms that drove and caused deviations in regional aridification and river flow played an important role in the diversification of the Yarkand hare. One possible interpretation of this result is that the living conditions for Yarkand hares were more favorable during the LGM than during the LIG period, perhaps because suitable habitat was reduced and thus range shifts occurred in the lowlands. However, extant populations have been confined to shrinking areas of suitable habitat (oases) during the present interglacial period. Our study sheds light on the impact of desert aridification and the associated influence of historical climate change on Yarkand hare diversification and phylogeography.

## Methods

### Field sampling and DNA extraction, amplification, and sequencing

The present study was conducted with permission and followed the animal research protocol IOZ-2006 approved by the Animal Care Committee of the Institute of Zoology, Chinese Academy of Sciences (IOZCAS). In total, 219 specimens of *L. yarkandensis* were collected from 21 locations (770–1330 m altitudes) throughout its distribution range within the oases of the Taklimakan Desert from 2001 to 2009 (Fig. 1b). The samples representing Yarkand hares are classified according to population boundaries/classification based on the previous studies. All the samples covered the southern, northern, eastern and western population in the Taklimakan Desert. The 21 sampling sites are elaborated in Table 2. All samples used in this study were collected from different sources, e.g., field surveys, road kill, and dried skins. No live animals were captured/sampled for the purposes of this study. The muscle tissues were collected and preserved in 100% ethanol. All skins and skulls collected in the field study were deposited in the mammal collections of the National Zoological Museum (NZM) of IOZCAS. The number of individuals sampled from the *L. yarkandensis* population is shown in Table 2.

**Table 2:** The number of individuals sampled by population of *Lepus yarkandensis* in Taklimakan Desert, China

Sampling site number	Location	Total number of individuals	Number of haplotypes	Distribution in Desert	Sample type	Stored in museum
1	Akesu	11	7	Northern population	Muscle	NZM of IOZ-CAS /GOM
2	Awati	10	8		Muscle	NZM of IOZ-CAS /GOM
3	Kuqa	9	7		Skin	NZM of IOZ-CAS /GOM
4	Shaya	8	4		Muscle	NZM of IOZ-CAS /GOM
5	Bugur	11	8		Muscle	NZM of IOZ-CAS /GOM
6	Marabishi	8	7	Western population	Muscle	NZM of IOZ-CAS /GOM
7	Kaxgar	7	5		Muscle	NZM of IOZ-CAS /GOM
8	Shule	11	4		Skin	NZM of IOZ-CAS /GOM
9	Markit	7	6		Muscle	NZM of IOZ-CAS /GOM
10	Yarkant	9	5		Muscle	NZM of IOZ-CAS /GOM
11	Qaghiliq	10	4		Muscle	NZM of IOZ-CAS /GOM
12	Pishan	14	12		Muscle	NZM of IOZ-CAS /GOM
13	Keriya	14	5	Southern Population	Muscle	NZM of IOZ-CAS /GOM
14	Qira	12	8		Muscle	NZM of IOZ-CAS /GOM
15	Hotan	10	7		Muscle	NZM of IOZ-CAS /GOM
16	Moyu	12	4		Muscle	NZM of IOZ-CAS /GOM
17	Minfeng	9	5		Muscle	NZM of IOZ-CAS /GOM
18	Qiemo	9	5		Muscle	NZM of IOZ-CAS /GOM
19	Korla	15	10	Eastern population	Muscle	NZM of IOZ-CAS /GOM
20	Yuli	14	7		Muscle	NZM of IOZ-CAS /GOM
21	Ruoqiang	9	4		Skull	NZM of IOZ-CAS /GOM

Total genomic DNA of Yarkand hares were extracted through a TIANamp Genomic DNA Kit (DP304; Tiangen Biotech, Beijing, China) based on the manufacturer’s standard protocols. One mitochondrial gene, cytochrome *b* (*Cytb*), and two nuclear genes, mechano-growth factor (*MGF*) and spectrin beta non-erythrocytic 1 (*SPTBN1*), were amplified via polymerase chain reaction (PCR). The primers and annealing temperatures are listed in Additional file 1: Table S1. Amplifications were carried out in 25 μL reactions with 25 ng of extracted Yarkand hare DNA (approximately 1 μL), 200 μM of respective dNTP, 0.2 μM of respective primer, 0.75 units of LA Taq polymerase and 2.5 μL of 10× buffer. The amplifications were performed as follows: an initial denaturation at 94 °C for 3 min, followed upon 35 cycles with denaturation at 94 °C for 30 s, annealing at 50–60 °C for 30 s, and polymerization at 72 °C for 1 or 2 min, and a final extension carried out at 72 °C for 10 min. The primers and annealing temperatures are listed in Additional file 1: Table S1**.** All PCR products were directly sequenced in both directions with an ABi 3100 automatic sequencer (Applied Biosystems, Foster City, California, USA) using the ABi PRISM BigDye Terminator Cycle Sequencing Ready Reaction Kit with AmpliTaq DNA polymerase (Applied Biosystems, Foster City, California, USA). All sequences were manually trimmed using BioEdit 7.2.5 [105]; the *nDNA* markers *MGF* and *SPTBN1* were phased in DNASP 5.10.01 [106] to further improve the quality of the sequences and analysis. All sequences were aligned by using the MUSCLE algorithm [107] implemented in MEGA 6 [108]. All sequences used in the present study were deposited into GenBank with the following accession numbers: *Cytb*, MH545002–MH545121; *MGF*, MG060661–MG660784; and *SPTBN1*, MG098874–MG98984 (Additional file 1: Table S2). We used *Cytb*, *MGF* and *SPTBN1* genes to calculate the genetic parameters of four populations and each clade (Table 1). The haplotype diversity (Hd) and nucleotide diversity (Pi) was calculated based on *mtDNA* and *nDNA* sequence datasets using DNASP 5.10.01 [106]. We also calculated the number of polymorphic (segregating) sites and the raggedness statistic calculated for each gene.

### Inferring phylogenetic relationships and haplotype networks

Bayesian inference (BI) analysis was employed to infer the phylogenetic relationships. jModelTest 2 [109] was used to find the best-fit model of base pair substitutions for the dataset based on the Bayesian information criterion (BIC). MrBayes 3.2.6 [110] was implemented with BI to assess the phylogenetic relationships among the samples, and gene trees were constructed based on combined loci *MGF* and *SPTBN1*. Three parallel runs of one cold and three heated Markov chain Monte Carlo (MCMC) chains were performed for 20 million or more generations. Trees were sampled every 1000 generations to generate convergence (SD < 0.01). The first 25% of the generations were discarded as a “burn-in”, and the posterior probability was estimated for the remaining generations. The trees were then illustrated in Figtree 1.3.1 [111]. The genealogical relationships among the haplotypes of the Yarkand hare were estimated by using median-joining (MJ) networks [112]. The final results were generated using the *mtDNA* sequences in PopART 1.7 [113].

### Divergence time estimation

To estimate the divergence time of the Yarkand hare, we used a Bayesian MCMC analysis in the BEAST 1.8.2 program [114]. The divergence time analysis was performed with *Cytb* because it possesses a higher degree of variation than the two nuclear genes [74, 115]. The clock model was chosen based on the marginal likelihood estimate (MLE) from stepping-stone and path sampling [116], with 100 path steps, 1,000,000 iterations, and sampling every 2000 generations. A HKY + I + G model of base pair substitution and an uncorrelated relaxed log-normal molecular clock with a prior coalescent tree of constant size were used. A normal distribution was employed, and default values were used for the other priors. The MCMC analysis was run twice for 20 million generations, with sampling every 1000 generations. The independent analyses were carried out based on the *Cytb* gene. We used Tracer 1.6 to examine the log files and check for stationarity and convergence of the chains to confirm that the effective sampling sizes (ESSs) for all parameters were greater than 200 [117]. We used the Tree annotator 1.8.2 in the BEAST package to summarize the tree data, with the first 25% of trees being discarded as a burn-in. The tree and divergence times were displayed and edited in Figtree 1.4.2 [23, 74]. We calibrated three points to build the time tree: i) the *Lepus* split around the divergence of *Silvilagus* at 8.61 MYA ii) the *L. hainanus* and *L. europaeus* split at 1.84 MYA; iii) the *L. yarkandensis* and *L. sinensis* split at 0.84 MYA [23]. Based on the previous work [23] numerous accurate fossil datasets were used to calibrate the evolutionary time of the Lepus genus. In previous research, the origin of Yarkand hare was at approximately 0.84 MYA [23]; therefore, we used this time as the divergence time for Yarkand hare. The selected outgroups are listed in Additional file 1: Table S3.

### Historical demographic changes

Population demography was estimated based on the *Cytb* because of the high level of genetic variations. Tajima’s *D* [65] and Fu’s *Fs* [118] analyses were carried out to test neutrality based on *Cytb* with 10,000 bootstrap replicates in Arlequin 3.5.2.2 [119] for the six clades. Fu’s *Fs* test carried out to detect population growth and genetic hitchhiking [118]. The mismatch distributions of pairwise sequence differences in Yarkand hare clades were estimated in Arlequin 3.5.2.2 [119] with 10,000 bootstrap replicates. Unimodal mismatch distributions and small Rag values with nonsignificant *p*-values for the sum of square deviation (SSD) imply recent demographic expansion or range expansion of a population [120]. To estimate the changes in effective population size (Ne) through evolutionary time, we also explored demographic history by constructing extended Bayesian skyline plots (EBSPs) in BEAST 1.8.2 with *Cytb* and two nuclear genes *MGF, SPTBN1*. For the parameters other than the prior tree, the settings were similar to those used in the divergence time analysis. The prior tree was set as the coalescent EBSP and sampled every 1000 steps for all 20 million steps. The MCMC convergence and ESS values for all the parameters were assessed in Tracer v.1.6.

### Species distribution modeling

We used species distribution models (SDMs) to predict the geographic distribution of suitable habitats for the Yarkand hare during different periods. We collected occurrence data from genetically identified specimens; locality datasets from field surveys; records from mammal collections of the NZM, IOZCAS; and previous studies [29, 61]. We obtained 19 climatic (bioclim) variables for the last interglacial (LIG), last glacial maximum (LGM) and current periods from the WorldClim v1.4 database (http://www.worldclim.org) [121]. Paleoclimate data were downloaded from the Community Climate System Model (CCSM) [122, 123], and the Model for Interdisciplinary Research on Climate (MIROC) [124] was used to carry out the analyses. We generated correlation matrices between the climatic variables to eliminate highly correlated variables from the models (Pearson’s r > 0.8); as a result, eight variables were included in the modeling: bio1 (annual mean temperature), bio4 (temperature seasonality), bio9 (mean temperature of driest quarter), bio11 (mean temperature of coldest quarter), bio12 (annual precipitation), bio13 (precipitation of wettest month), bio18 (precipitation of warmest quarter), and bio19 (precipitation of coldest quarter). We included a total of 19 climatic variables, and 8 of these variables are common and reliable variables [125, 126]. The current distributions were modeled using Maxent 3.3.3 k [127, 128].

The Maxent model was projected with the past climatic conditions to characterize the past distribution. We used default settings for parameters such as prevalence, regularization multiplier [129], and density of background sampling (10,000 points) but created multiple replicate models and explored the implications of different combinations of climatic variables. We ran models with 10 cross-validated replicates by randomly assigning the presence records to training and test datasets (90 and 10%, respectively). We assessed the performance of the models by calculating classification errors using the area under the receiver operating characteristic curve (AUC), sensitivity, specificity, and true skill statistic (TSS = sensitivity + specificity - 1) for each of the 12 models that were cross-validated against the training dataset [130–133] [29, 62–64]. We used 75% of the data to calibrate the models and 25% to calculate performance efficiency. Finally, the models with high TSS values were chosen to generate the final distribution models (AUC > 0.8 and TSS > 0.6). To perform this analysis, we used ArcGIS version 10.2 [134].

### Spatial diffusion analysis

To examine the origin and range expansion of the Yarkand hare and test the hypothesis of isolation through time, we carried out phylogeographical diffusion analysis (GEO_SPHERE package) in BEAST 2.4.7 [135]. To attain better convergence, we reduced the dataset to one individual per haplotype per set of geographic coordinates. A total of 99 haplotypes and 21 sets of geographic coordinates were used based on the format prescribed in [136]. The divergence time was calibrated with the divergence points of six main clades estimated from the BEAST tree analysis for 3,000,000 generations, with sampling every 3000 generations. The convergence of runs and thus the support for the inferred ages of migration events was achieved by ensuring that the ESS for the “geotreelikelihood” prior was greater than 200 in the log file. The time slicer in SPREAD v1.0.6 [136] was employed to view the spatiotemporal diffusion pattern. The final results were visualized in Google Earth (Google, California, USA, available at http://google.com/earth/).

## Additional files

Additional file 1: **Table S1.** Primers and PCR cycling conditions for three genes used to reconstruct the phylogenetic structure of *Lepus yarkandensis* in Taklimakan Desert, China. **Table S2.** GenBank accession numbers with sample information for individuals used in this study. **Table S3.** The following sequences used for the time tree analysis. **Table S4.** Genetic diversity and neutrality test estimates of *Lepus yarkandensis* based on *MGF* loci. **Table S5.** Genetic diversity and neutrality test estimates of *Lepus yarkandensis* based on *SPTBN1* loci. (DOCX 42 kb)

Additional file 2: **Figure S1.** Phylogenetic tree of *MGF* & *SPTBN1* combined from the Bayesian inference analysis. The Bayesian posterior probabilities are shown on the branches. **Figure S2.** The distribution ranges of *Lepus yarkandensis* and *Lepus capensis* based on IUCN Red List assessment. **Figure S3. (a)** Pairwise mismatch distributions for entire sequences of *mtDNA* **(b)** nuclear genes *MGF* and **(c)** *SPTBN1.* The coloured bars indicates the observed distribution of pairwise differences, and black dashed lines represent the theoretical expected distribution under a population expansion model. (**d**) The historical demographic trends represented by Extended Bayesian Skyline Plot (EBSP) based on all the sequences of *Cytb*. **(e)** nuclear genes *MGF* and **(f)** *SPTBN1.*The median population size in the blue coloured lines, and the red lines representing the upper and lower 95% confidence intervals. The x-axis of the skyline plots is time in millions of years before the present, and they y–axis is the estimated effective population size (*Ne*). **Figure S4. (a)** The Median-joining network of MGF gene and **(b)** SPTBN1 network tree for *Lepus yarkandensis*. The colouring is based on the four populations. The shared haplotypes names are indicated on the network tree. The circles represent individual haplotypes with size proportional to frequency, branches indicate mutations and black circles indicates hypothetical ancestors. Dots represent undetected haplotypes. The names of the shared haplotypes are indicated on the network tree. (DOCX 827 kb)

## Abbreviations

AUC: Area under the receiver operating characteristic curve
BI: Bayesian inference
BIC: Bayesian information criterion
CCSM: Community Climate System Model
Cytb: Cytochrome B
EBSP: Extended Bayesian Skyline Plot
ESS: Effective Sampling Size
GOM: Group Of Mammalogy
IOZ-CAS: Institute of Zoology- Chinese Academy of Sciences
IUCN: International Union for Conservation of Nature
Ka: Kilo-Annum
LGM: Last Glacial Maxima
LIG: Last Inter Glacial
MCMC: Markov Chain Monte Carlo
MGF: Mechano Growth Factor
MIROC: The Model for Interdisciplinary Research on Climate
MJ: Median-joining
MLE: Marginal Likelihood Estimate
MMD: Mis Match Distribution
mtDNA: Mitochondrial DNA
MTS: Mountains
MYA: Million Years Ago
nDNA: Nuclear DNA
NE: Population Size
NT: Near Threatened
NZM: National Zoological Museum
QTP: Qinghai Tibetan Plateau
SDM: Species Distribution Model
SPTBN1: Spectrin Beta nonerythrocytic 1
SSD: Sum of Square Deviation
TSS: True skill statistic
