# Supplementary material for: Phylogeography and ecological niche modeling unravel the evolutionary history of the Yarkand hare, Lepus yarkandensis (Mammalia: Leporidae), through the Quaternary
Source: BMC Evol Biol. 2019 Jun 1;19:113. doi: 10.1186/s12862-019-1426-z (PMC6545225; doi:10.1186/s12862-019-1426-z)
Supplement: Supplementary file 1 — Table S1. Primers and PCR cycling conditions for three genes used to reconstruct the phylogenetic structure of Lepus yarkandensis in Taklimakan Desert, China. Table S2. GenBank accession numbers with sample information for individuals used in this study. Table S3. The following sequences used for the time tree analysis. Table S4. Genetic diversity and neutrality test estimates of Lepus yarkandensis based on MGF loci. Table S5. Genetic diversity and neutrality test estimates of Lepus yarkandensis based on SPTBN1 loci. (DOCX 42 kb) [file 12862_2019_1426_MOESM1_ESM.docx]

**Additional file 1**

**Phylogeography and ecological niche modeling unravel the evolutionary history of the Chinese endemic Yarkand hare, *Lepus yarkandensis* (Mammalia:** **Leporidae), through the Quaternary**

**Brawin Kumar^1,2^, Jilong Cheng^1^, Deyan Ge^1^, Lin Xia^1^, Qisen Yang^1^**

**Table S1** Primers and PCR cycling conditions for three genes used to reconstruct the phylogenetic structure of *Lepus yarkandensis* in Taklimakan Desert, China.

| Locus | Base Pair Length | Primer sequence | | | Annealing temperature (℃) | References |
| --- | --- | --- | --- | --- | --- | --- |
| Code |  | Forward (5’ – 3’) | Reverse (5’ – 3’) | | |  |
| *Cytb* | 1,140 | CAACTACAAGAACCTAATGACCAA | CAGGGTAATAYACTATACTACTGG | | 51 | [1] |
| *Mgf* | 592 | AAATATCAGTCTTGAATCTTAC | | TTTTAGATGAATTACAGTGTCC | 54 | [2] |
| *Sptbn* | 619 | CTCTGCCCAGAAGTTTGCAAC | TGATAGCAGAACTCCATGTGG | | 55 | [2] |

**Table S2.** GenBank accession numbers with sample information for individuals used in this study

| S.No | Taxon | Sampling location | Haplotype | Genbank Accession Number |
| --- | --- | --- | --- | --- |
|  |  |  | Cytb |  |
| 1 | *Lepus yarkandensis* | AKESU01 AKESU12 | Hap1 | MH545002 |
| 2 | *Lepus yarkandensis* | AKESU02 YULI2 YULI5 YULI8 YULI16 YULI17 KUQA4 KUQA8 | Hap2 | MH545003 |
| 3 | *Lepus yarkandensis* | AKESU03 AKESU10 | Hap3 | MH545004 |
| 4 | *Lepus yarkandensis* | AKESU04 AKESU07 MARBAISHI4 MARBAISHI5 YULI7 YULI18 QIEMO8 SHAYA4 SHULE7 SHULE10 KERIYA1 KERIYA4 KERIYA6 KERIYA7 KERIYA8 | Hap4 | MH545005 |
| 5 | *Lepus yarkandensis* | AKESU05 AKESU09 YULI20 | Hap5 | MH545006 |
| 6 | *Lepus yarkandensis* | AKESU06 | Hap6 | MH545007 |
| 7 | *Lepus yarkandensis* | AKESU08 AKESU11 | Hap7 | MH545008 |
| 8 | *Lepus yarkandensis* | AKESU13 | Hap8 | MH545009 |
| 9 | *Lepus yarkandensis* | AKESU14 AKESU17 MARBAISHI13 MARBAISHI14 KUQA6 KUQA9 | Hap9 | MH545010 |
| 10 | *Lepus yarkandensis* | AKESU15 | Hap10 | MH545011 |
| 11 | *Lepus yarkandensis* | PISHAN01 YARKAND4 | Hap11 | MH545012 |
| 12 | *Lepus yarkandensis* | AKESU18 | Hap12 | MH545013 |
| 13 | *Lepus yarkandensis* | AKESU19 | Hap13 | MH545014 |
| 14 | *Lepus yarkandensis* | MARBAISHI1 MARBAISHI19 KORLA7 | Hap14 | MH545015 |
| 15 | *Lepus yarkandensis* | MARBAISHI2 MARBAISHI10 MARBAISHI12 MARBAISHI18 YARKAND6 | Hap15 | MH545016 |
| 16 | *Lepus yarkandensis* | MARBAISHI3 YULI9 | Hap16 | MH545017 |
| 17 | *Lepus yarkandensis* | MARBAISHI6 MARBAISHI8 MARBAISHI9 KORLA1 KORLA6 KORLA8 KORLA15 KORLA17 | Hap17 | MH545018 |
| 18 | *Lepus yarkandensis* | MARBAISHI7 KAXGAR4 KAXGAR19 | Hap18 | MH545019 |
| 19 | *Lepus yarkandensis* | MARBAISHI11 QAGHILIQ3 | Hap19 | MH545020 |
| 20 | *Lepus yarkandensis* | MARBAISHI15 | Hap20 | MH545021 |
| 21 | *Lepus yarkandensis* | MARBAISHI16 | Hap21 | MH545022 |
| 22 | *Lepus yarkandensis* | MARBAISHI17 | Hap22 | MH545023 |
| 23 | *Lepus yarkandensis* | MARBAISHI20 KORLA4 | Hap23 | MH545024 |
| 24 | *Lepus yarkandensis* | QIRA1 QIRA2 QIRA5 QIRA6 QIRA8 QIRA10 QIRA16 QIRA18 | Hap24 | MH545025 |
| 25 | *Lepus yarkandensis* | QIRA3 QIRA7 QIRA14 QIRA17 HOTEN9 HOTEN10 HOTEN11 HOTEN12 PISHAN02HOTEN17 BUGUR8 BUGUR11 BUGUR14 BUGUR18 MOYU4 MOYU5 MOYU6 MOYU7 MOYU8 MOYU12 KAXGAR13 KAXGAR22 RUOQIANG1 RUOQIANG2 RUOQIANG4 RUOQIANG6 | Hap25 | MH545026 |
| 26 | *Lepus yarkandensis* | QIRA4 QIRA15 HOTEN6 HOTEN7 HOTEN8 | Hap26 | MH545027 |
| 27 | *Lepus yarkandensis* | QIRA9 RUOQIANG3 RUOQIANG10 | Hap27 | MH545028 |
| 28 | *Lepus yarkandensis* | QIRA11 | Hap28 | MH545029 |
| 29 | *Lepus yarkandensis* | QIRA12 | Hap29 | MH545030 |
| 30 | *Lepus yarkandensis* | QIRA13 | Hap30 | MH545031 |
| 31 | *Lepus yarkandensis* | QIRA19 | Hap31 | MH545032 |
| 32 | *Lepus yarkandensis* | QIRA20 | Hap32 | MH545033 |
| 33 | *Lepus yarkandensis* | HOTEN1 | Hap33 | MH545034 |
| 34 | *Lepus yarkandensis* | HOTEN2 HOTEN3 YULI6 | Hap34 | MH545035 |
| 35 | *Lepus yarkandensis* | HOTEN4 HOTEN5 BUGUR4 BUGUR7 YULI16 | Hap35 | MH545036 |
| 36 | *Lepus yarkandensis* | HOTEN13 | Hap36 | MH545037 |
| 37 | *Lepus yarkandensis* | HOTEN14 | Hap37 | MH545038 |
| 38 | *Lepus yarkandensis* | HOTEN15 | Hap38 | MH545039 |
| 39 | *Lepus yarkandensis* | HOTEN18 | Hap39 | MH545040 |
| 40 | *Lepus yarkandensis* | HOTEN19 | Hap40 | MH545041 |
| 41 | *Lepus yarkandensis* | HOTEN20 MOYU1 PISHAN03 AWAT04 | Hap41 | MH545042 |
| 42 | *Lepus yarkandensis* | YULI1 | Hap42 | MH545043 |
| 43 | *Lepus yarkandensis* | YULI3 | Hap43 | MH545044 |
| 44 | *Lepus yarkandensis* | YULI4 | Hap44 | MH545045 |
| 45 | *Lepus yarkandensis* | YULI6 YULI15 | Hap45 | MH545046 |
| 46 | *Lepus yarkandensis* | YULI10 | Hap46 | MH545047 |
| 47 | *Lepus yarkandensis* | YULI11 KUQA2 KUQA11 | Hap47 | MH545048 |
| 48 | *Lepus yarkandensis* | YULI12 | Hap48 | MH545049 |
| 49 | *Lepus yarkandensis* | YULI13 | Hap49 | MH545050 |
| 50 | *Lepus yarkandensis* | YULI14 | Hap50 | MH545051 |
| 51 | *Lepus yarkandensis* | BUGUR1 BUGUR13 BUGUR15 | Hap51 | MH545052 |
| 52 | *Lepus yarkandensis* | BUGUR2 BUGUR17 | Hap52 | MH545053 |
| 53 | *Lepus yarkandensis* | BUGUR3 BUGUR9 BUGUR16 YULI2 YULI10 YULI13 YULI26 | Hap53 | MH545054 |
| 54 | *Lepus yarkandensis* | BUGUR5 | Hap54 | MH545055 |
| 55 | *Lepus yarkandensis* | BUGUR6 | Hap55 | MH545056 |
| 56 | *Lepus yarkandensis* | BUGUR10 MINGFENG1 MINGFENG2 MINGFENG3 MINGFENG5 MINGFENG6 MINGFENG7 MINGFENG8 MINGFENG9 PISHAN04 MINGFENG11 MINGFENG14 MINGFENG17 MINGFENG18 MINGFENG19 MINGFENG20 MINGFENG21 RUOQIANG7 AWAT03 | Hap56 | MH545057 |
| 57 | *Lepus yarkandensis* | BUGUR12 | Hap57 | MH545058 |
| 58 | *Lepus yarkandensis* | KORLA2 KORLA16 | Hap58 | MH545059 |
| 59 | *Lepus yarkandensis* | KORLA3 | Hap59 | MH545060 |
| 60 | *Lepus yarkandensis* | KORLA5 KAXGAR2 KAXGAR18 KAXGAR21 KAXGAR24 AWAT02 | Hap60 | MH545061 |
| 61 | *Lepus yarkandensis* | KORLA9 | Hap61 | MH545062 |
| 62 | *Lepus yarkandensis* | KORLA10 | Hap62 | MH545063 |
| 63 | *Lepus yarkandensis* | KORLA11 YARKAND2 | Hap63 | MH545064 |
| 64 | *Lepus yarkandensis* | KORLA12 | Hap64 | MH545065 |
| 65 | *Lepus yarkandensis* | KORLA13 | Hap65 | MH545066 |
| 66 | *Lepus yarkandensis* | KORLA14 | Hap66 | MH545067 |
| 67 | *Lepus yarkandensis* | MINGFENG4 | Hap67 | MH545068 |
| 68 | *Lepus yarkandensis* | MINGFENG12 | Hap68 | MH545069 |
| 69 | *Lepus yarkandensis* | MINGFENG13 | Hap69 | MH545070 |
| 70 | *Lepus yarkandensis* | MINGFENG15 | Hap70 | MH545071 |
| 71 | *Lepus yarkandensis* | MINGFENG16 | Hap71 | MH545072 |
| 72 | *Lepus yarkandensis* | MINGFENG22 | Hap72 | MH545073 |
| 73 | *Lepus yarkandensis* | MINGFENG23 | Hap73 | MH545074 |
| 74 | *Lepus yarkandensis* | MINGFENG24 MINGFENG25 MINGFENG27 RUOQIANG1 RUOQIANG2 RUOQIANG3 RUOQIANG4 RUOQIANG8 YULI12 YULI19 YULI22 | Hap74 | MH545075 |
| 75 | *Lepus yarkandensis* | MARABISHI1 RUOQIANG5 | Hap75 | MH545076 |
| 76 | *Lepus yarkandensis* | MOYU2 MOYU11 | Hap76 | MH545077 |
| 77 | *Lepus yarkandensis* | MOYU3 MOYU9 KAXGAR5 KAXGAR11 YULI14 | Hap77 | MH545078 |
| 78 | *Lepus yarkandensis* | KAXGAR1 KAXGAR6 KAXGAR7 KAXGAR9 KAXGAR15 KAXGAR23 KAXGAR25 | Hap78 | MH545079 |
| 79 | *Lepus yarkandensis* | KAXGAR3 KAXGAR12 | Hap79 | MH545080 |
| 80 | *Lepus yarkandensis* | KAXGAR8 KAXGAR14 | Hap80 | MH545081 |
| 81 | *Lepus yarkandensis* | KAXGAR10 | Hap81 | MH545082 |
| 82 | *Lepus yarkandensis* | KAXGAR16 | Hap82 | MH545083 |
| 83 | *Lepus yarkandensis* | KAXGAR17 | Hap83 | MH545084 |
| 84 | *Lepus yarkandensis* | KAXGAR20 | Hap84 | MH545085 |
| 85 | *Lepus yarkandensis* | QIEMO1 QIEMO2 AWAT01 RUOQIANG9 YULI5 | Hap85 | MH545086 |
| 86 | *Lepus yarkandensis* | QIEMO3 QIEMO6 | Hap86 | MH545087 |
| 87 | *Lepus yarkandensis* | QIEMO4 | Hap87 | MH545088 |
| 88 | *Lepus yarkandensis* | QIEMO5 | Hap88 | MH545089 |
| 89 | *Lepus yarkandensis* | QIEMO7 | Hap89 | MH545090 |
| 90 | *Lepus yarkandensis* | RUOQIANG6 | Hap90 | MH545091 |
| 91 | *Lepus yarkandensis* | YARKAND1 | Hap91 | MH545092 |
| 92 | *Lepus yarkandensis* | YARKAND3 | Hap92 | MH545093 |
| 93 | *Lepus yarkandensis* | YARKAND5 | Hap93 | MH545094 |
| 94 | *Lepus yarkandensis* | SHAYA1 | Hap94 | MH545095 |
| 95 | *Lepus yarkandensis* | SHAYA2 | Hap95 | MH545096 |
| 96 | *Lepus yarkandensis* | SHAYA3 PISHAN05 RUOQIANG8 | Hap96 | MH545097 |
| 97 | *Lepus yarkandensis* | SHAYA5 | Hap97 | MH545098 |
| 98 | *Lepus yarkandensis* | SHULE1 SHULE11 | Hap98 | MH545099 |
| 99 | *Lepus yarkandensis* | SHULE2 SHULE14 | Hap99 | MH545100 |
| 100 | *Lepus yarkandensis* | SHULE3 SHULE4 SHULE6 SHULE8 SHULE9 SHULE12 | Hap100 | MH545101 |
| 101 | *Lepus yarkandensis* | MARABISHI2 MARABISHI3 | Hap101 | MH545102 |
| 102 | *Lepus yarkandensis* | KUQA1 KUQA12 | Hap102 | MH545103 |
| 103 | *Lepus yarkandensis* | KUQA3 | Hap103 | MH545104 |
| 104 | *Lepus yarkandensis* | KUQA5 | Hap104 | MH545105 |
| 105 | *Lepus yarkandensis* | KUQA7 | Hap105 | MH545106 |
| 106 | *Lepus yarkandensis* | KUQA10 | Hap106 | MH545107 |
| 107 | *Lepus yarkandensis* | KERIYA2 KERIYA5 | Hap107 | MH545108 |
| 108 | *Lepus yarkandensis* | KERIYA3 | Hap108 | MH545109 |
| 109 | *Lepus yarkandensis* | KERIYA9 | Hap109 | MH545110 |
| 110 | *Lepus yarkandensis* | KERIYA10 | Hap110 | MH545111 |
| 111 | *Lepus yarkandensis* | QAGHILIQ1 | Hap111 | MH545112 |
| 112 | *Lepus yarkandensis* | QAGHILIQ2 | Hap112 | MH545113 |
| 113 | *Lepus yarkandensis* | QAGHILIQ4 | Hap113 | MH545114 |
| 114 | *Lepus yarkandensis* | QAGHILIQ5 | Hap114 | MH545115 |
| 115 | *Lepus yarkandensis* | YULI1 YULI3 | Hap115 | MH545116 |
| 116 | *Lepus yarkandensis* | YULI4 YULI23 | Hap116 | MH545117 |
| 117 | *Lepus yarkandensis* | YULI7 | Hap117 | MH545118 |
| 118 | *Lepus yarkandensis* | YULI8 | Hap118 | MH545119 |
| 119 | *Lepus yarkandensis* | YULI9 YULI11 YULI15 YULI18 YULI24 YULI25 | Hap119 | MH545120 |
| 120 | *Lepus yarkandensis* | YULI17 MARABISHI4 | Hap120 | MH545121 |

**Table S3** The following sequences used for the time tree analysis.

| Species name | Gen Bank Number |
| --- | --- |
| *Lepus americanus* | AY292733.1 |
| *Lepus brachyurus* | AB058616.2 |
| *Lepus californicus* | AY292731.1 |
| *Lepus capensis* | NC_015841.1 |
| *Lepus comus* | AJ279408.1 |
| *Lepus europaeus* | NC_004028.1 |
| *Lepus hainanus* | AY745114.1 |
| *Lepus mandshuricus* | DQ793162.1 |
| *Lepus oiostolus* | AY599081.1 |
| *Lepus saxatilis* | AY292730.1 |
| *Lepus sinensis* | AJ279419.1 |
| *Lepus timidus* | AJ279424.1 |
| *Lepus townsendii* | AY292729.1 |

| S.No | Name of the Location | Population in Desert | Gene/Loci | Number of polymorphic (segregating) sites, S: | Total number of mutations, Eta: | Haplotype (gene) diversity, Hd: | Nucleotide diversity, Pi: | R Stat | Tajima’s *D* | Fu’s *Fu* |
| --- | --- | --- | --- | --- | --- | --- | --- | --- | --- | --- |
| 1 | Akesu | Northern | MGF | 6 | 6 | 0.945 | 0.00697 | 0.1055 | 0.68508 | -3.577 |
| 2 | Awati |  | MGF | 5 | 4 | 0.774 | 0.00339 | 0.0422 | 0.25441 | -3.222 |
| 3 | Kuqa |  | MGF | 5 | 2 | 0.972 | 0.00857 | 0.0378 | -0.68469 | -2.69 |
| 4 | Shaya |  | MGF | 5 | 2 | 1 | 0.00592 | 0.0889 | 0.04995 | -2.898 |
| 5 | Bugur |  | MGF | 5 | 2 | 1 | 0.01122 | 0.0255 | -0.28376 | -3.657 |
| 6 | Marabishi | Western | MGF | 4 | 3 | 1 | 0.00805 | 0.0998 | -0.25712 | -3.18 |
| 7 | Kaxgar |  | MGF | 5 | 2 | 0.933 | 0.00583 | 0.0933 | -1.25192 | -1.009 |
| 8 | Shule |  | MGF | 5 | 2 | 1 | 0.00579 | 0.2778 | 0.17969 | -1.082 |
| 9 | Markit |  | MGF | 5 | 2 | 1 | 0.00767 | 0.0952 | 0.09834 | -3.232 |
| 10 | Yarkant |  | MGF | 4 | 1 | 1 | 0.00722 | 0.0309 | 0.1386 | -5.556 |
| 11 | Qaghiliq |  | MGF | 5 | 1 | 1 | 0.00761 | 0.2578 | -0.24625 | -2.518 |
| 12 | Pishan |  | MGF | 5 | 1 | 0.956 | 0.00825 | 0.5381 | 0.2687 | -2.063 |
| 13 | Keriya | Southern | MGF | 4 | 0 | 0 | 0 | 0 | 0 | 0 |
| 14 | Qira |  | MGF | 5 | 0 | 0.889 | 0.00677 | 0.1481 | -0.50521 | -0.531 |
| 15 | Hotan |  | MGF | 5 | 1 | 0.8333 | 0.00719 | 0.4167 | 0.37186 | 0.646 |
| 16 | Moyu |  | MGF | 5 | 1 | 1 | 0.00981 | 0.0544 | 0.13759 | -2.639 |
| 17 | Mingfeng |  | MGF | 5 | 3 | 0.756 | 0.00496 | 0.1289 | 0.05917 | 0.218 |
| 18 | Qiemo |  | MGF | 5 | 1 | 1 | 0.00893 | 0.0711 | 0.64755 | -2.035 |
| 19 | Korla | Eastern | MGF | 6 | 2 | 1 | 0.00959 | 0.0499 | 0 | -2.695 |
| 20 | Yuli |  | MGF | 8 | 3 | 1 | 0.00694 | 0.12 | 0.29358 | -1.716 |
| 21 | Ruoqiang |  | MGF | 8 | 4 | 1 | 0.00938 | 0.2844 | -0.24351 | -1.987 |

**Table S4.** Genetic diversity and neutrality test estimates of *Lepus yarkandensis* based on *MGF* loci.

**Table S5.** Genetic diversity and neutrality test estimates of *Lepus yarkandensis* based on *SPTBN1* loci.

| S.No | Name of the Location | Population in Desert | Gene/Loci | Number of polymorphic (segregating) sites, S: | Total number of mutations, Eta: | Haplotype (gene) diversity, Hd: | Nucleotide diversity, Pi: | R Stat | Tajima’s *D* | Fu’s *Fu* |
| --- | --- | --- | --- | --- | --- | --- | --- | --- | --- | --- |
| 1 | Akesu | Northern | SPTBN1 | 6 | 4 | 0.673 | 0.00505 | 0.0879 | -0.34669 | -0.29 |
| 2 | Awati |  | SPTBN1 | 5 | 4 | 1 | 0.00611 | 0.5 | 0.6118 | -0.388 |
| 3 | Kuqa |  | SPTBN1 | 5 | 6 | 0.929 | 0.01024 | 0.1939 | -1.26961 | -1.98 |
| 4 | Shaya |  | SPTBN1 | 5 | 15 | 0.933 | 0.03279 | 0.2 | -1.22536 | 2.165 |
| 5 | Bugur |  | SPTBN1 | 5 | 1 | 0.571 | 0.00231 | 0.3469 | 1.34164 | 0.856 |
| 6 | Marabishi | Western | SPTBN1 | 4 | 2 | 0.733 | 0.00344 | 0.3467 | -0.05002 | -0.427 |
| 7 | Kaxgar |  | SPTBN1 | 5 | 9 | 1 | 0.00712 | 0.2 | 0.4606 | -1.481 |
| 8 | Shule |  | SPTBN1 | 5 | 5 | 1 | 0.00598 | 0.5 | -0.75445 | -2.367 |
| 9 | Markit |  | SPTBN1 | 5 | 4 | 0.9 | 0.00906 | 0.19 | -1.38419 | -0.773 |
| 10 | Yarkant |  | SPTBN1 | 4 | 8 | 1 | 0.00115 | 0.0648 | -0.16987 | -7.178 |
| 11 | Qaghiliq |  | SPTBN1 | 5 | 9 | 1 | 0.00782 | 0.1667 | 0.52223 | -0.48 |
| 12 | Pishan |  | SPTBN1 | 5 | 11 | 1 | 0.03025 | 0.0638 | -1.56448 | -0.935 |
| 13 | Keriya | Southern | SPTBN1 | 4 | 7 | 1 | 0.00866 | 0.0355 | 0.5227 | 0.884 |
| 14 | Qira |  | SPTBN1 | 5 | 12 | 1 | 0.00829 | 0.0357 | 0.63535 | -3.566 |
| 15 | Hotan |  | SPTBN1 | 5 | 4 | 0.9 | 0.00547 | 0.0374 | 0.08857 | 0.177 |
| 16 | Moyu |  | SPTBN1 | 5 | 9 | 1 | 0.00075 | 0.558 | 0.03584 | 0.596 |
| 17 | Mingfeng |  | SPTBN1 | 5 | 1 | 0.429 | 0.00178 | 0.2041 | 0.3335 | 0.536 |
| 18 | Qiemo |  | SPTBN1 | 5 | 6 | 0.9 | 0.00499 | 0.19 | 0.08298 | -0.128 |
| 19 | Korla | Eastern | SPTBN1 | 6 | 13 | 1 | 0.0094 | 0.16 | 0.18621 | -0.964 |
| 20 | Yuli |  | SPTBN1 | 8 | 9 | 1 | 0.00764 | 0.22 | -0.10905 | -1.283 |
| 21 | Ruoqiang |  | SPTBN1 | 8 | 1 | 0.667 | 0.00098 | 0.5556 | 0.2553 | -0.487 |
