# Supplementary material for: Phylogeography and ecological niche modeling unravel the evolutionary history of the Yarkand hare, Lepus yarkandensis (Mammalia: Leporidae), through the Quaternary
Source: BMC Evol Biol. 2019 Jun 1;19:113. doi: 10.1186/s12862-019-1426-z (PMC6545225; doi:10.1186/s12862-019-1426-z)
Supplement: Supplementary file 2 — Figure S1. Phylogenetic tree of MGF & SPTBN1 combined from the Bayesian inference analysis. The Bayesian posterior probabilities are shown on the branches. Figure S2. The distribution ranges of Lepus yarkandensis and Lepus capensis based on IUCN Red List assessment. Figure S3. (a) Pairwise mismatch distributions for entire sequences of mtDNA (b) nuclear genes MGF and (c) SPTBN1. The coloured bars indicates the observed distribution of pairwise differences, and black dashed lines represent the theoretical expected distribution under a population expansion model. (d) The historical demographic trends represented by Extended Bayesian Skyline Plot (EBSP) based on all the sequences of Cytb. (e) nuclear genes MGF and (f) SPTBN1.The median population size in the blue coloured lines, and the red lines representing the upper and lower 95% confidence intervals. The x-axis of the skyline plots is time in millions of years before the present, and they y–axis is the estimated effective population size (Ne). Figure S4. (a) The Median-joining network of MGF gene and (b) SPTBN1 network tree for Lepus yarkandensis. The colouring is based on the four populations. The shared haplotypes names are indicated on the network tree. The circles represent individual haplotypes with size proportional to frequency, branches indicate mutations and black circles indicates hypothetical ancestors. Dots represent undetected haplotypes. The names of the shared haplotypes are indicated on the network tree. (DOCX 827 kb) [file 12862_2019_1426_MOESM2_ESM.docx]

**Additional file 2**

**Phylogeography and ecological niche modeling unravel the evolutionary history of the Chinese endemic Yarkand hare, *Lepus yarkandensis* (Mammalia:** **Leporidae), through the Quaternary**

**Brawin Kumar^1,2^, Jilong Cheng^1^, Deyan Ge^1^, Lin Xia^1^, Qisen Yang^1^**


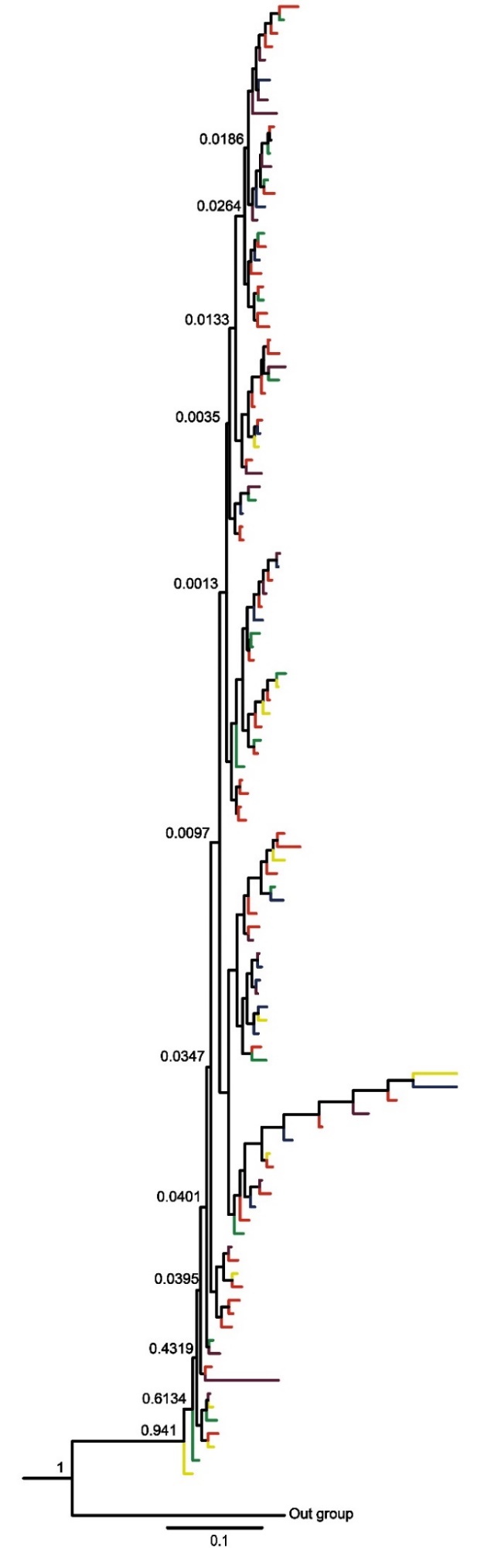


**Figure S1** Phylogenetic tree of *MGF* & *SPTBN1* combined from the Bayesian inference analysis. The Bayesian posterior probabilities are shown on the branches.

**
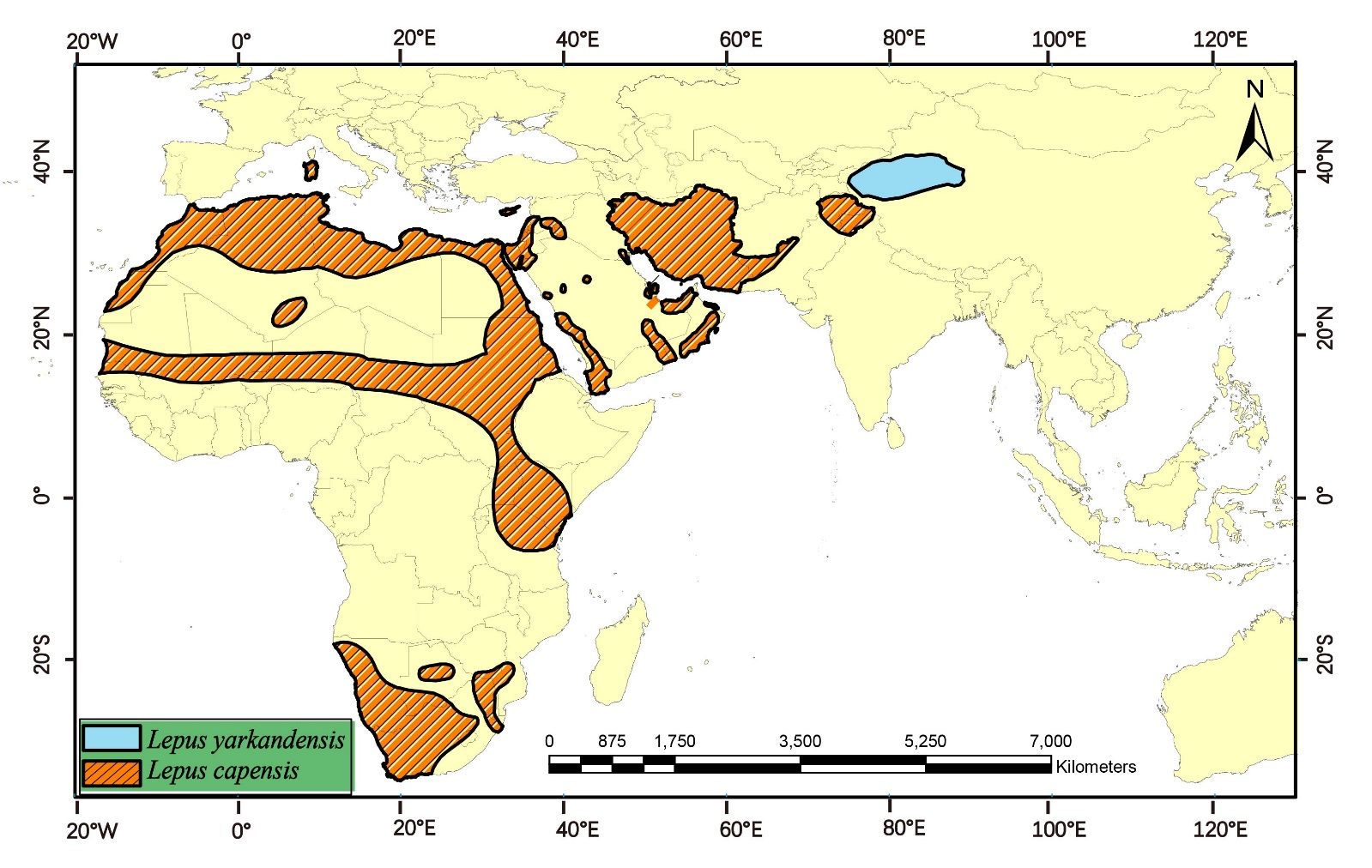
Figure S2** The distribution ranges of *Lepus yarkandensis* and *Lepus capensis* based on IUCN Red List assessment.

**
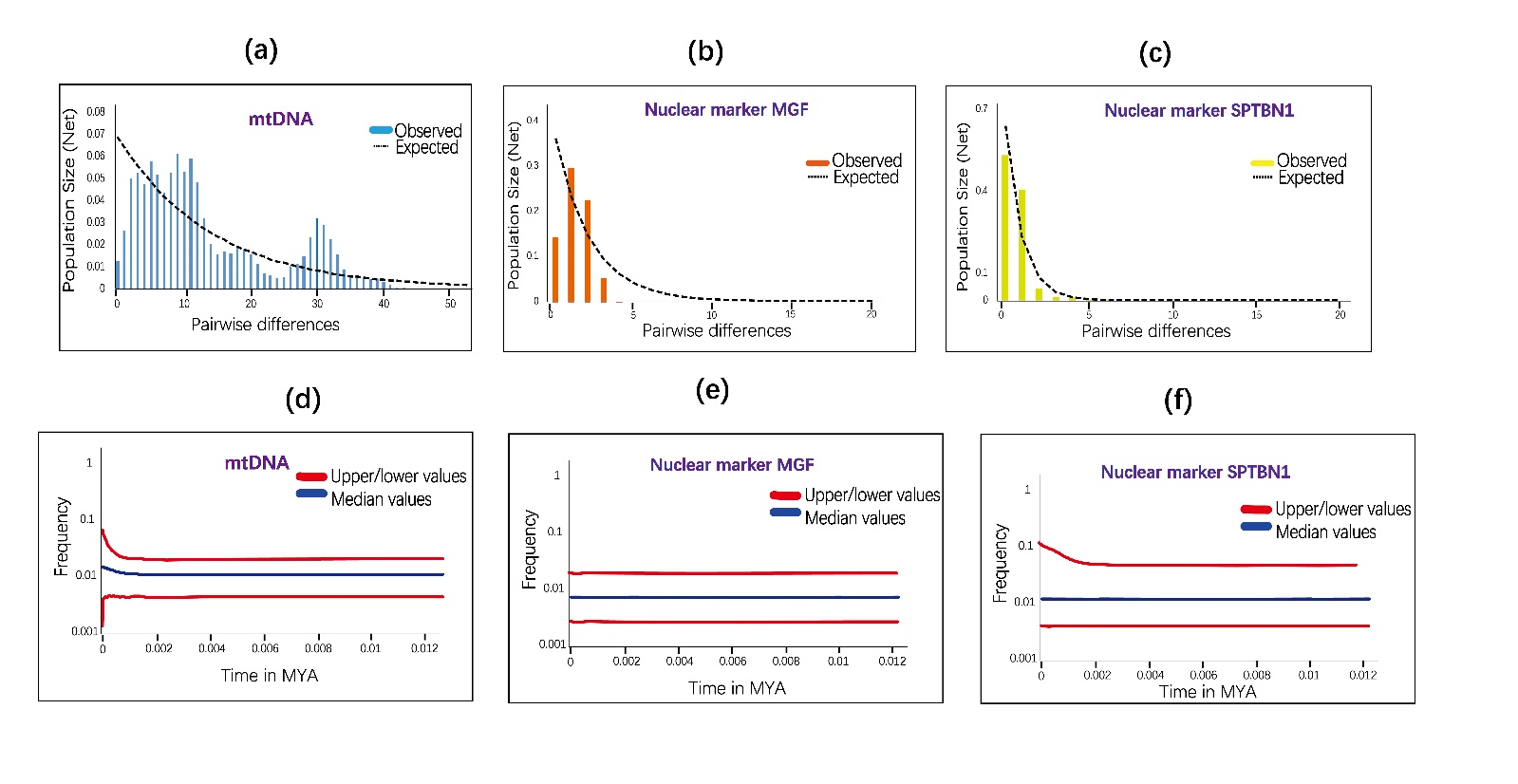
**

**Figure S3 (a)** Pairwise mismatch distributions for entire sequences of *mtDNA* **(b)** nuclear genes *MGF* and **(c)** *SPTBN1.* The coloured bars indicates the observed distribution of pairwise differences, and black dashed lines represent the theoretical expected distribution under a population expansion model. (**d**) The historical demographic trends represented by Extended Bayesian Skyline Plot (EBSP) based on all the sequences of *Cytb*. **(e)** nuclear genes *MGF* and **(f)** *SPTBN1.*The median population size in the blue coloured lines, and the red lines representing the upper and lower 95% confidence intervals. The x-axis of the skyline plots is time in millions of years before the present, and they y–axis is the estimated effective population size (*Ne*).

**
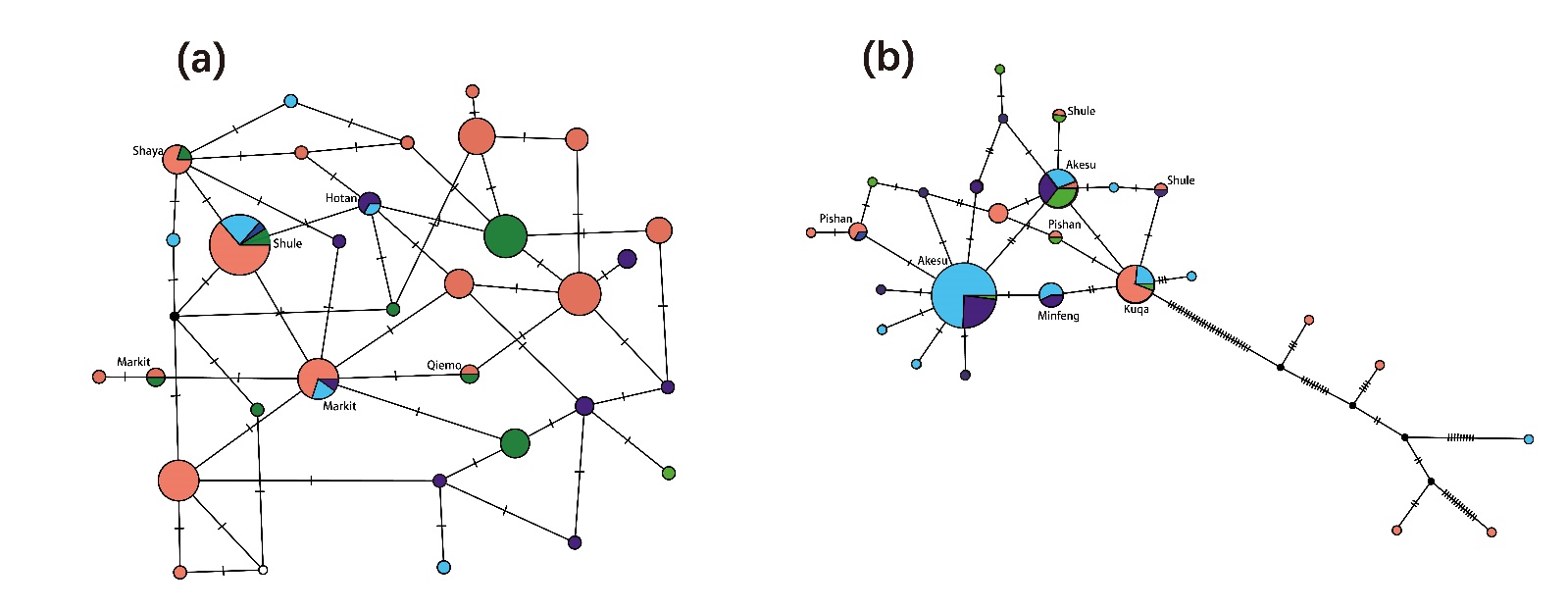
**

**Figure S4 (a)** The Median-joining network of MGF gene and **(b)** SPTBN1 network tree for *Lepus yarkandensis*. The colouring is based on the four populations. The shared haplotypes names are indicated on the network tree. The circles represent individual haplotypes with size proportional to frequency, branches indicate mutations and black circles indicates hypothetical ancestors. Dots represent undetected haplotypes. The names of the shared haplotypes are indicated on the network tree.

**References**

1. Liu J, Yu L, Arnold ML, Wu CH, Wu SF, Lu X, Zhang YP. Reticulate evolution: frequent introgressive hybridization among chinese hares (genus *lepus*) revealed by analyses of multiple mitochondrial and nuclear DNA loci. BMC Evol Biol*.* 2011;11:223.

2. Matthee CA, Van Vuuren BJ, Bell D, Robinson TJ, Sullivan J. A molecular supermatrix of the rabbits and hares (Leporidae) allows for the identification of five intercontinental exchanges during the Miocene. Syst Biol. 2004;53:433–447.
